# Introgression of Black Rot Resistance from *Brassica carinata* to Cauliflower (*Brassica oleracea botrytis* Group) through Embryo Rescue

**DOI:** 10.3389/fpls.2017.01255

**Published:** 2017-07-18

**Authors:** Brij B. Sharma, Pritam Kalia, Dinesh Singh, Tilak R. Sharma

**Affiliations:** ^1^Division of Vegetable Science, Indian Council of Agricultural Research-Indian Agricultural Research Institute New Delhi, India; ^2^Division of Plant Pathology, Indian Council of Agricultural Research-Indian Agricultural Research Institute New Delhi, India; ^3^National Agri-Food Biotechnology Institute Mohali, India

**Keywords:** Cole crops, *Xanthomonas campestris* pv. *campestris*, resistance breeding, inter-specific hybridization, ovule culture, intron length polymorphic markers

## Abstract

Black rot caused by *Xanthomonas campestris* pv. *campestris* (*Xcc*) is a very important disease of cauliflower (*Brassica oleracea botrytis* group) resulting into 10–50% yield losses every year. Since there is a dearth of availability of resistance to black rot disease in *B. oleracea* (C genome), therefore exploration of A and B genomes was inevitable as they have been reported to be potential reservoirs of gene(s) for resistance to black rot. To utilize these sources, interspecific hybrid and backcross progeny (B_1_) were generated between cauliflower “Pusa Sharad” and Ethiopian mustard “NPC-9” employing *in vitro* embryo rescue technique. Direct ovule culture method was better than siliqua culture under different temperature regime periods. Hybridity testing of F_1_ inter-specific plants was carried out using co-dominant SSR marker and *Brassica* B and C genome-specific (DB and DC) primers. Meiosis in the di-genomic (BCC) interspecific hybrid of *B. oleracea botrytis* group (2*n* = 18, CC) × *B. carinata* (2*n* = 4x = 34, BBCC) was higly disorganized and cytological analysis of pollen mother cells revealed chromosomes 2*n* = 26 at metaphase-I. Fertile giant pollen grain formation was observed frequently in interspecific F_1_ hybrid and BC_1_ plants. The F_1_ inter-specific plants were found to be resistant to *Xcc* race 1. Segregation distortion was observed in BC_1_ generation for black rot resistance and different morphological traits. The At1g70610 marker analysis confirmed successful introgression of black rot resistance in interspecific BC_1_ population. This effort will go a long way in pyramiding gene(s) for resistance against black rot in Cole crops, especially cauliflower and cabbage for developing durable resistance, thus minimize dependency on bactericides.

## Introduction

Cauliflower (*Brassica oleracea botrytis* group, 2*n* = 18, C genome) is one of the extensively grown *Brassica* vegetables around the world. Black rot caused by the gram negative bacterium *Xanthomonas campestris* pv. *campestris* (Pammel) Dowson is one of the most threating diseases in vegetable *Brassicas* worldwide (Vicente and Holub, 2013). It has adverse effect on quality and yield losses up to 10–50% have been reported under appropriate environmental conditions in cauliflower (Singh et al., 2011). The disease spreads through vascular tissues, clogging vessels and producing V-shaped chlorotic lesions. Managing the disease is very difficult as the bacterium spreads within and between fields by water splashes, wind, insects, machinery and irrigation. Although chemicals for controlling the diseases are available, but deployment of resistant cultivars is reliable, economical and environmentally safe approach to manage this disease. Periodic outbreaks of the black rot disease have occurred worldwide, especially in the developing countries of Africa and Asia, where high temperatures and humidity favor the disease (Hayward, 1993). Although nine pathogenic races of *Xcc* have been identified (Tonu et al., 2013), but race 1 and 4 are predominant worldwide. Recently *Xcc* races 1, 4, and 6 were identified in India and, among these race 1 followed by race 4 dominated in most of the states (Singh et al., 2016). Resistance to these two races (race 1 and 4) did not exist or was very rare, in contrast to common resistance to less important races (2, 3, and 6) in *B. oleracea*. Race specific resistance to races 1 and/or 4 is frequently found in other species of the genus *Brassica* (Taylor et al., 2002). Exploring the new resistance sources in alien *Brassica* species and its introgression into *B. oleracea* group is one of the current priority areas for black rot resistance breeding. The black rot resistance sources have been reported in A and B genomes of *Brassica* species (Westman et al., 1999; Taylor et al., 2002) and genes present in these genomes can be pyramided in Cole crops to provide a durable solution to this malady. The monogenomic and digenomic species in Uthant triangle carry resistance gene(s) for *Xcc*, which can be utilized for guarding *B. oleracea* genotypes against this disease using embryo rescue and somatic hybridization techniques. Genetic transformation option can be explored when resistance is not available within and related gene pools. Besides, although, a number of disease resistance genes in *Brassicas* and *A. thaliana* have been postulated and mapped, but none has been cloned as yet (Vicente and Holub, 2013). Therefore, there is no information on genetic transformation for resistance to black rot disease in *B. oleracea* group.

*Brassica carinata A. Braun* (Ethiopian mustard, 2*n* = 4x = 34, BBCC genome) is an important oilseed crop thought to have originated in the Ethiopian plateau (Warwick, 2011). Its leaves are also consumed as vegetable in North East Africa. The *Brassica* B genome is known to carry several important traits, yet there has been limited analyses of its underlying genome structure (Navabi et al., 2013). *B. carinata* harbors genes controlling valuable resistance/tolerance traits for biotic and abiotic stresses viz., resistance to black leg, black rot and tolerance to aluminum, salinity, heat and drought (Pan et al., 2012; Enjalbert et al., 2013). Moreover, strong resistance to *Xcc* race 1 and 4 has been reported in high proportion of *B. nigra* (B genome) and *B. carinata* (BC genome) accessions (Taylor et al., 2002; Vicente et al., 2002). A single dominant gene conferring high resistance to *Xcc* race 1 was reported by Tonguc et al. (2003). Recently, a single dominant gene (*Xca1bc*) controlling black rot resistance was mapped with intron length polymorphic markers at B-7 genome of *B. carinata* (Sharma et al., 2016). Therefore, it is important and feasible to transfer this black rot resistance gene into cauliflower cultivars. Alien gene transfer in crop plants has emerged as a potent approach to broaden gene pools and increase genetic variability in existing germplasm. Hybridization between a diploid and a tetraploid species is difficult and failure occurs at many stages starting from pollination incompatibility to pre/post-fertilization barriers.

Search for new gene(s) for disease resistance in closer genome related species viz., *B. napus* (AACC) and *B. carinata* (BBCC) for incorporation into *B. oleracea* group via interspecific (Tonguc et al., 2003) or somatic hybridization using embryo rescue (Hansen and Earle, 1995) has to be a continuous process. Of the three *Brassica* genomes, A and C are very closely related while the B-genome is phylogenetically distant. Zhou et al. (1997) transferred resistance to *Xcc* from *B. napus* to broccoli (*B. oleracea*) through protoplast fusion. Tonguc and Griffiths (2004b), however, explored the half B genome *B. juncea* (AABB) for the introgression of black rot resistance into *B. oleracea* using *in vitro* embryo rescue. Somatic hybridization was attempted to transfer black rot resistance from *B. nigra* into *B. oleracea* (Wang et al., 2011, 2016).

Utilization of embryo rescue or somatic hybridization techniques, or combination of both, could help to overcome natural reproductive barriers in the development of inter-specific hybrids in *Brassica* (Ayotte et al., 1987; Hansen and Earle, 1995; Momotaz et al., 1998; Weerakoon et al., 2009; Niemann et al., 2013). Wide crosses between crop plants and their wild relatives have now become routinely possible through the use of the embryo rescue technique. Different techniques of plant cell and tissue culture, such as ovary, ovule and embryo culture as well as protoplast fusion, have proved useful for production of interspecific hybrids. Rescue of hybrid embryos and their culture *in vitro* helps to overcome post-fertilization barriers in interspecific crosses. Since its first use in *Brassica* by Nishi et al. (1959), extensive investigations have been carried out to improve the techniques for obtaining higher seed set (Inomata, 1993, 2002; Zhang et al., 2003, 2004). The successful application of this technique depends on the stage of the embryo being rescued and cultured *in vitro*. Several attempts were made to transfer desirable gene(s) from alien *Brassica* spp. to *B. oleracea* such as powdery mildew resistance (Tonguc and Griffiths, 2004a), downy mildew (Chiang et al., 1977), male sterility (Chiang and Crete, 1987) and atrazine resistance (Jourdan et al., 1989). The progress for marker assisted *Xcc* resistance gene transfer from *B. carinata* to cauliflower has been very slow (Tonguc et al., 2003; Tonguc and Griffiths, 2004b). The present study was carried out to transfer a monogenic resistance to *Xcc* race 1 from an accession “NPC-9” of *B. carinata* into a commercial susceptible cauliflower variety “Pusa Sharad” using embryo rescue technique.

## Materials and methods

### Development of plant materials

**Figure 1:**
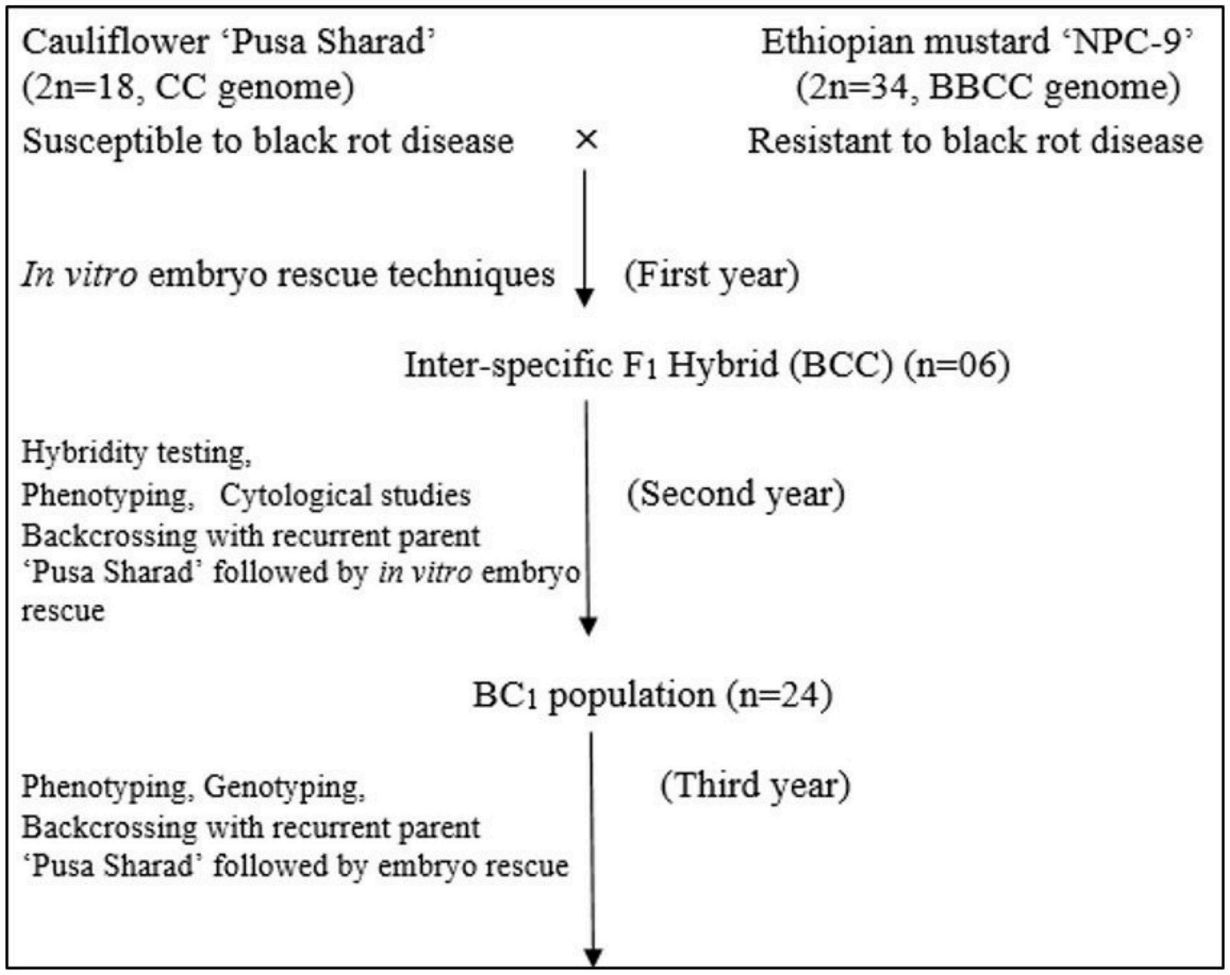
Flow chart of development of plant materials using *in vitro* embryo rescue technique.

Inbred of black rot susceptible variety “Pusa Sharad” of cauliflower (*Brassica oleracea botrytis* group, 2*n* = 18, C) was crossed with a resistant accession “NPC-9” (*Brassica carinata* A. Braun; 2*n* = 4x = 34, BC genome) maintained by repeated selfing. The susceptible parent “Pusa Sharad” was planted sequentially from end of September to November 2011 at Vegetable Research Farm, ICAR- Indian Agricultural Research Institute, New Delhi. Seeds of “NPC-9” were sown in the end of October 2011, 2 months later than the recurrent parent Pusa Sharad to ensure synchronous flowering. Fully developed unopened flower buds which are expected to open within next 1–2 days were selected for pollination. These flower buds were emasculated and pollinated on the day of anthesis with fresh pollen grains collected from freshly opened flowers of donor parent. Nearly 10–15 flower buds were pollinated in each inflorescence to ensure better seed set. Simultaneously, the other flower buds which have not been pollinated were removed to avoid any unwanted seed set. All plants were covered with selfing bags to avoid any out crossing and pollen contamination. After pollination, the pollinated buds were covered with butter paper bags for 5–6 days to prevent out crossing. Development of plant materials is explained in flow diagram (Figure 1).

### *In vitro* embryo rescue

Effect of days after pollination on siliqua and ovule culture were studied under two temperature regime viz. low temperature (Day/Night, 19.4°C/4.3°C) and High temperature (Day/Night, 26.4°C/10.4°C). The cross combination “Pusa Sharad × NPC-9” was advanced *in vitro* through immature siliqua and direct ovule culture. A total of 480 immature siliqua were harvested from 5 to 21 DAP at 2 days interval for the rescue of hybrid embryos. Harvested siliqua were placed in a 100 ml conical flask washed by tap water and then surface sterilized with 0.1% HgCl_2_ solution for 1 min followed by 4–5 times thorough washings with double distilled sterile water. The siliqua were then sterilized with 70% alcohol for 5–7 min and followed by 2–3 wash with doubled distilled sterile water. For siliqua culture, parts of the ovary stalks were transversely cut on autoclaved filter paper in petri dishes and the ovaries were placed with the basal cut end on the media. Siliqua culture medium was Murashige and Skoog's (1962) supplemented with 3% (w/v) sucrose, 7% agar and casein hydrolysate (400 mg/L) at pH = 5.8, autoclaved for 22 min at 121°C. The siliqua culture was maintained in culture room at 25 ± 2°C having automatically set timings with white fluorescent lights under a 16/8 h (light/dark) photoperiod. The cultured siliqua were carefully dissected aseptically 5–15 days later and enlarged/turgid ovules were cultured on basal MS medium supplemented with 3% (w/v) sucrose and kept in the dark until germination.

In direct ovule culture method, hybrid siliqua were harvested from 11 to 21 DAP at 2 days interval. Ovules from the siliquae were carefully dissected on filter paper in petri dishes aseptically and cultured on basal MS medium with 6% (w/v) sucrose and were kept in dark until germination. Sub culturing of the ovules was done on the same medium at 15 days interval. Putative hybrid plantlets were multiplied *in vitro* via shoot tip culture on MS medium with 1 mg BAP l^−1^, 3% (w/v) sucrose.Well-developed plantlets were transferred on MS medium with 0.5 mg IBA L^−1^ for *in vitro* rooting. *In vitro* hardening of roots was done by keeping bare roots in test tubes filled with tap water and covered with polythene for 7–10 days. Well established 3–4 week old seedlings were transferred in the plastic pots filled with solarite (Keltech Company, Maharashtra, India) and covered with polythene bags for acclimatization. All plastic pots were placed in shade house for acclimatization under natural photoperiod followed by transplanting in the earthen pots filled with pot mixture (Garden soil + FYM+ Sand) which were then shifted to open field conditions for advancing growing plants till maturity. The two embryo rescue methods (siliqua and ovule culture) employed under two temperature regimes were statistically analyzed using *t*-test (Gupta and Kapoor, 2002).

### Production of BC_1_ plants

The F_1_ interspecific hybrid plants were grown under open field conditions during 2012–13. The highly resistant F_1_ individual plants were backcrossed with recipient recurrent parent “Pusa Sharad” of cauliflower and the BC_1_ plants were generated through direct ovule culture method of embryo rescue. Protocol for direct ovule culture and plantlets regeneration was same as described earlier for F_1_ production. BC_1_ plants were grown under natural open conditions during 2013–14.

### Hybridity confirmation

Dominant morphological anther tip pigmentation marker was used to confirm hybridity of interspecific F_1_ plants. B genome specific 30 SSR markers were retrieved from publicly available data bank in the *Brassica* microsatellite information exchange (www.brassica.info/resource/markers/ssr-exchange.php). *Brassica* B and C genome specific primers DB and DC (Supplementary Table 1) were used for interspecific hybridity testing as described by Yan et al. (2014).

### Morphological characterization

The parental genotypes, F_1_ and BC_1_ plants were characterized for different 20 morphological traits comprising eleven leaf related trait, viz. leaf attitude, length (cm), width (cm), shape, lobes, color, waxiness, puckering, leaf apex, mid vein thickness and dentations of margins and one for plant height (cm), eight floral and siliqua related traits like color of petals, stalk length (cm), stalk color, petals length (cm), width of petals (cm), siliqua length (cm), siliqua angel with main shoot and siliqua texture. The measurements of these parameters were taken by adopting characters described in DUS guideline of Ethiopian mustard and cauliflower (http://www.plantauthority.gov.in/crop-guidelines.htm)

### DNA extraction and PCR conditions

The DNA was isolated from the young leaf tissues obtained from the parents, F_1_ and BC_1_ plants using Cetyl trimethyl ammonium bromide (CTAB) method (Murray and Thompson, 1980). The DNA was then purified and quantified on 0.8% agarose gel by comparison with 50 ng/μl of standard uncut lambda (λ) DNA marker. DNA was diluted in sterile distilled water to a concentration of 25–30 ng μl^−1^. SSR analysis was carried out in which PCR reactions were set up in 0.2 ml thin walled sterilized PCR tubes. Amplifications were carried out in a total volume of 15 μl reaction volume containing 1.5 μl 10X PCR assay buffer with 17.5 mM MgCl_2_, 0.6 μl dNTP mix (10 Mm of each dATP, dTTP, dCTP, and dGTP for direct use in PCR), 0.2 μl *Taq* DNA polymerase (5U μl^−1^), primer (10 μM) 1.5 μl each forward and reverse, 3 μl template DNA (25–30 ng) and sterile water (6.70 μl). All the PCR chemicals were obtained from Hi media Laboratories, Mumbai, India. The PCR amplification reaction was carried out in a thermocycler (Eppendorf master cycler) with the initial PCR cycle temperature conditions of 94°C for 5 min for DNA denaturation followed by 35 cycles of 45 s each for denaturation at 94°C, primer annealing at 50–65°C varying with primer-pairs for 1 min, 2 min extension at 72°C and final extension at 72°C for 7 min.

PCR conditions for DB and DC primers were carried out with the initial PCR cycle at 94°C for 3 min for DNA denaturation followed by 34 cycles of 30 s denaturation at 94°C, primer annealing at 58–60°C for 30 s, 2 min extension at 72°C and final extension at 72°C for 8 min.The amplified products were resolved by electrophoresis in 3% agarose (Hi media product, India) gel containing ethidium bromide in 1X TAE buffer (pH 8.0) at constant voltage (@5v/cm) for 3h. The size of the amplified fragment was determined by co-electrophoresis of standard molecular weight marker. DNA profile was visualized on UV transilluminator and photographed by using gel documentation System (Alpha Imager, Cell Bioscience).

Genotyping of BC_1_ population was carried out using linked ILPAt1g70610 marker for black rot resistance (Sharma et al., 2016). This analysis was carried out by using PCR conditions as initial denaturation at 94°C for 5 min, followed by 35 cycles of denaturation at 94°C for 1 min annealing at 54.9°C for 45 s and elongation at 72°C for 3 min followed by a final extension at 72°C for 10 min. Amplified PCR products were analyzed on 2% w/v agarose gel.

### Cytological studies

For the study of meiotic behavior in the interspecific hybrids, immature flower buds of different size (2.5–4.5 mm) were collected from F_1_ interspecific hybrid. Meiotic chromosome preparations were carried out as described by Leflon et al. (2006) and Mason et al. (2010), that is, the buds were fixed in carnoy's solution (ethanol: chloroform: acetic acid, 6:3:1) for 24 h at room temperature (25°C) and stored in 50% ethanol at 4°C. Anthers were squashed and stained in a drop of 1% acetocarmine solution. The slides were examined under a phase contrast microscope and chromosome counting was observed in 10 pollen mother cells (PMCs) per plant at metaphase I. The pollen grains from three flowers from each of the genotypes viz. Pusa Sharad, NPC-9, each of inter-specific F_1_ hybrid plants and all the BC_1_ plants were stained with 1% acetocarmine to know pollen viability status. Approximately, 100 pollen grains from each of the three flowers were observed for staining under the microscope. The percentage of stainable pollen grains was calculated to measure pollen fertility. Pollen size was measured under Zeiss stereo microscope with Axio Cam 1Cc-1 camera. Giant pollen was defined as pollen with more than 1.5 × normal diameter (Mason et al., 2011).

### Artificial inoculation of *Xcc* and disease assessment

The interspecific F_1_s and backcross segregating generation (B_1_) were screened against *Xcc* race 1 during November to December 2012 and 2013, respectively. The mean monthly weather data for experimental period (October, 2012–March, 2014) were collected from Division of Agriculture Physics, ICAR-IARI, New Delhi. Average maximum (21.7°C–27.3°C) and minimum temperature (7.5°C–9.9°C) with high relative humidity (84.2–89%) and low rainfall (0.3 mm) were recorded during phenotyping period (November–December, 2012). During the year 2013 (November–December), the average maximum and minimum temperature ranged from 22.4°C-26.9°C, 7.1°C-9.9°C, respectively with high relative humidity (90.9–93.7%) and low rainfall (0.2 mm). Besides, the experimental pots were watered frequently during the period of inoculation to maintain high humidity required for proper disease development. These agrometeorological conditions favored disease establishment and development during the phenotyping period.

The bacterial strain (Accession number, ITCC-BH-0001; Delhi isolates, C1) of *Xcc* race 1 (Singh et al., 2016) was obtained from the Bacteriology Unit, Division of Plant Pathology, ICAR-IARI, New Delhi, India and multiplied in yeast glucose chalk agar (YGCA) media at 25°C for 3 days. The culture was carefully scrapped from the media with sterilized slide. The scraped bacterial culture was mixed in 100 ml sterilized distilled water and mixed thoroughly by vortex and final concentration of 10^8^–10^9^ cfu/ml was made. The plants were first inoculated on 30th day after planting by using leaf cut and dip technique (Kapoor et al., 1985) by clipping the secondary veins at the margins with small scissor dipped in the bacterial suspension. The inoculation was carried out at 10 points per leaf on youngest leaves in three replications. Three observations were recorded at weekly interval and final disease scoring was done at 30 DAI.The inoculated plants were assessed for disease reaction based on disease rating scale of 0–9 and percentage of inoculated points in leaves showing symptoms were recorded and grouping of plants were done into resistant and susceptible categories in segregating populations according to Vicente et al. (2002). The total number of inoculated points and the number of points showing symptoms were recorded and the percentage of infected points were calculated termed as percent disease index (PDI). The severity of symptoms was assessed on a six-point scale of 0–9 based on the relative lesion size (0, no symptoms; 1, small necrosis or chlorosis surrounding the infection point; 3, typical small V-shaped lesion with black veins; 5, typical lesion half way to the middle vein; 7, typical lesion progressing to the middle vein; and 9, lesion reaching the middle vein). Generally, plants with a score of 0, 1, or 3 with 0 to 25% (PDI) points showing symptoms were considered resistant; plants with a score of 3 with more than 25% of points showing symptoms and those with a score of 5 with less than 50% of points showing symptoms were considered partially resistant; plants with a score of 5 with more than 50% of points showing symptoms and plants with a score of 7 with less than 75% of points showing symptoms were considered susceptible, whereas plants with a score of 7 with more than 75% of points showing symptoms and with a score of 9 with 100% points showing symptoms were considered very susceptible.

## Results

### Embryo rescue

A successful response in terms of interspecific F_1_ hybrid and BC_1_ generation development were obtained in cross combination “Pusa Sharad × NPC-9” (Tables 1, 2). Under natural conditions, the siliqua were enlarged but all the ovules were shriveled/dried at 25 DAP leading to no seed formation. We followed two methods of *in vitro* embryo rescue, namely siliqua and ovule culture. From siliqua culture, 101 turgid ovules were obtained by culturing 240 immature siliqua. On cutting open of these siliqua after 15 days of *in vitro* culture, all ovules were shriveled and brown which did not germinate, whereas 5–7 days after, enlarged/turgid ovules could be excised from *in vitro* cultured siliqua. During low temperature regime (19.4°C/4.3°C), 7–11 DAP stage was identified optimum for *in vitro* siliqua culture producing maximum number (36) of turgid ovules. Two of which only germinated from which one grew up as true hybrid. Very slow growth of hybrid siliqua was observed due to low temperature prevalence in natural open conditions. During high temperature regime, 5–7 DAP stage of hybrid siliqua was found optimum for siliqua culture. However, some of the ovaries ceased growing at later stage and their embryos degenerated. Even single turgid ovule could not be obtained from 15 DAP. Out of 30 turgid ovules, only one germinated and produced hybrid. The success rate of siliqua culture method was slightly higher (2.12%) at low temperature regime (19.4°C/4.3°C) than high temperature (26.4°C/10.4°C) regime (1.85%).

**Table 1:** Effect of days after pollination on siliqua and ovule culture of inter-specific hybrid of *Brassica oleracea botrytis* group and *Brassica carinata* and under different temperature regime.

**Embryo rescue method**	DAP^*^	**Low temperature regime day/night (19.4°C/4.3°C)**				**High temperature regime day/night (26.4°C/10.4°C)**				**Total true hybrid**	^*^Average success rate (%)
		**Turgid ovules**	**Germinated ovules**	**True hybrid**	**Success rate (%)**	**Turgid ovules**	**Germinated ovules**	**True hybrid**	**Success rate (%)**		
Siliqua culture	5	04	0	0	0	13	0	0	0	0	–
	7	13	0	0	0	17	01	01	5.88	01	–
	9	15	01	01	6.67	10	0	0	0	01	–
	11	08	01	0	0	08	0	0	0	0	–
	13	05	0	0	0	06	0	0	0	0	–
	15	02	0	0	0	0	0	0	0	0	–
Total	–	47	02	01	2.12	54	01	01	1.85	02	1.98
Ovule culture	11	06	0	0	0	16	01	01	6.25	01	–
	13	08	0	0	0	21	03	02	9.52	02	–
	15	13	1	0	0	15	01	0	0	0	–
	17	19	03	01	5.26	11	0	0	0	01	–
	19	10	0	0	0	04	0	0	0	0	–
	21	01	0	0	0	01	0	0	0	0	–
*t*-test		−0.51	−0.62	0.11	–	−0.18	−1.14	−0.52	–	−0.32	
*p*-value		0.62	0.54	0.91	–	0.86	0.27	0.61	–	0.75	
Total		57	04	01	1.75	68	04	03	4.41	04	3.20

* Success rate percentage (No. of hybrid plants obtained/ No. of turgid ovules cultured) × 100 %, DAP^*^, Days after pollination.

**Table 2:** Production of BC_1_ plants using direct ovule culture under two different temperature regimes.

DAP^*^	**Low temperature regime day/night (19.4°C/4.3°C)**				**High temperature regime day/night (26.4°C/10.4°C)**				**Total No. of BC1 plants**	^*^Average success rate (%)
	**Turgid ovules**	**Germinated ovules**	**No. of BC_1_ plants**	**Success rate (%)**	**Turgid ovules**	**Germinated ovules**	**No. of BC_1_ plants**	**Success rate (%)**		
11	06	0	0	0	27	09	05	18.51	05	–
13	09	0	0	0	58	13	09	15.51	09	–
15	18	02	01	5.55	37	06	03	8.10	04	–
17	41	05	04	12.19	09	02	0	0	04	–
19	28	04	02	7.14	06	0	0	0	02	–
21	10	01	0	0	01	0	0	0	0	–
*t*-test^**^	2.58	1.78	1.95	–	2.05	2.16	1.77	–	2.43	
*p*-value	0.02	0.09	0.06	–	0.05	0.04	0.09	–	0.02	
Total	112	12	07	6.25	138	30	17	12.31	24	9.60

* Success rate percentage (No. of hybrid plants obtained/ No. of turgid ovules cultured) × 100 %, DAP*, Days after pollination.

** *Comparison of direct ovule culture for BC_1_ vs. direct ovule culture for F_1_ production under two different temperature regimes*.

In case of ovule culture method, the maximum numbers of turgid ovules (125) were obtained by culturing 240 ovaries. During low temperature regime, 42 turgid ovules were obtained at 15–19 DAP stage of embryo rescue. Out of these, 4 ovules germinated and hybrid seedling could be produced at a 17 DAP stage. In high temperature regime, 52 turgid ovules were obtained at 11–15 DAP and 05 of these germinated, two of which grew as hybrid plants. The siliqua and ovule culture embryo rescue methods under two temperature regimes were found to be statistically at par. However, the success rate in direct ovule culture method was higher (4.14%) at high temperature regime than low temperature regime period (1.75%). The average success rate of direct ovule culture method was higher (3.20%) than siliqua culture (1.98%). Therefore, direct ovule culture method was followed to produce first backcross generation (BC_1_) in the present study. Total of 24 BC_1_individuals were produced out of 42 germinated turgid ovules. This again proved that 17–19 DAP and 11–13 DAP were best time for direct ovule culture during low (19.4°C/4.3°C) than high (26.4°C/10.4°C) temperature regime periods as given in Table 2.

### Confirmation of inter-specific hybrids

**Figure 2:**
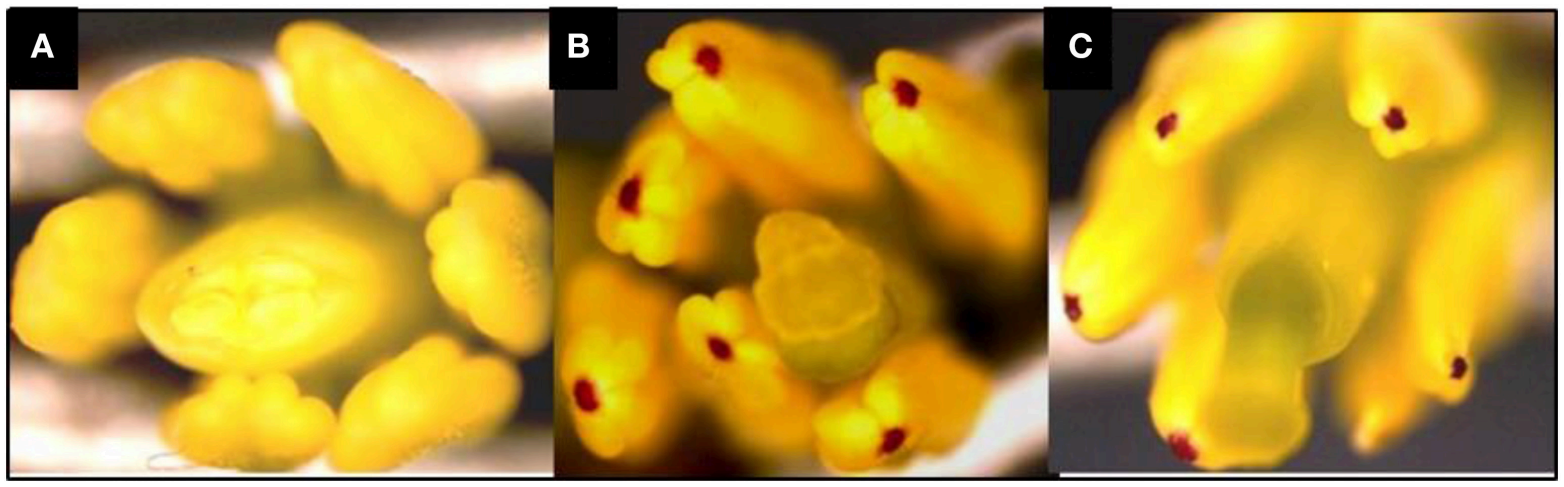
Anthocynin pigmentation on anther tip, a dominant morphological marker used in inter-specific hybridity testing. **(A)** Pigmentation absent in *B. oleracea botrytis* group “Pusa Sharad.” **(B)** Pigmentation present in *B. carinata* “NPC-9.” **(C)** Present in F_1_'s of Pusa Sharad × NPC-9.

**Figure 3:**
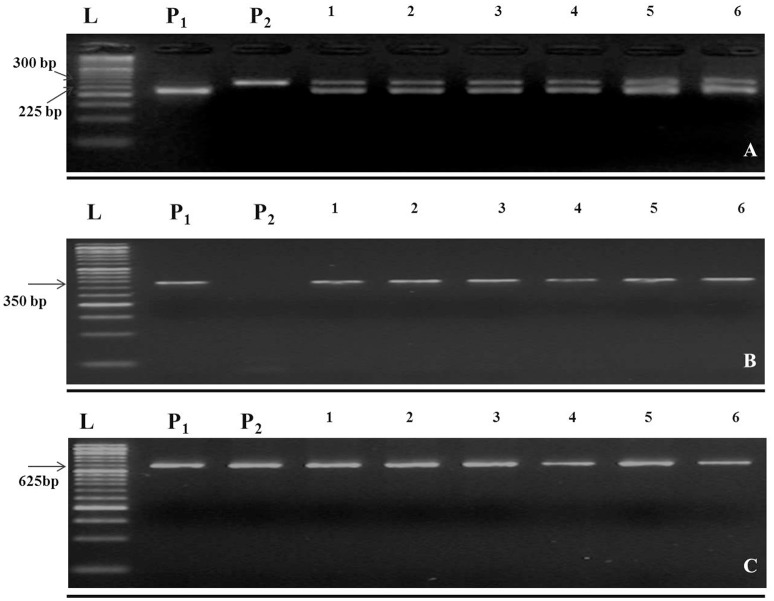
Molecular markers used in inter-specific hybridity testing of *B. oleracea botrytis* group “Pusa Sharad” (C genome) and *B. carinata* “NPC-9” (BC genome). **(A)** Codominant SSR NI2-C01. **(B)** B genome specific primer (DB) amplified 350 bp size. **(C)** C genome specific primer (DC) amplified 625 bp. P_1_; NPC-9 and P_2_; Pusa Sharad, 1-6; F_1_ individuals, M = 50 bp DNA ladder. (**A–C** were grouped together and separated using black horizontal line).

In this study, the recurrent parent “Pusa Sharad” variety of cauliflower did not show any pigmentation on anther tip. The *B. carinata* accession “NPC-9,” a donor parent, however, had anthocyanin pigmentation on anther tip. Presence of anthocyanin pigmentation on all F_1_ plants anther tip confirmed its hybridity (Figure 2). Thirty SSR markers were employed to find polymorphism between “Pusa Sharad” and “NPC-9.” From these, SSRNI2-C01 was chosen to confirm hybridity of F_1_ plants. This co-dominant marker generated ~225 bp fragment in *B. carinata* “NPC-9,” 300 bp fragment in *B. oleracea botrytis* group “Pusa Sharad” and both size fragments were found in all F_1_ interspecific plants confirming true hybridity (Figure 3A). Genome-specific amplification confirmed hybridity on using *Brassica TTG1* genome-specific DB and DC primers (Figures 3B,C). B genome specific primer (DB) amplified 350 bp size band in *Brassica carinata* (BBCC) as well as in all digenomic (BCC) F_1_ plants, while it did not amplify in the C genome (*B. oleracea. botrytis* group). The C genome specific primer (DC) amplified 625 bp size bands in both parents as well as in all F_1_ plants.

### Cytological studies

**Figure 4:**
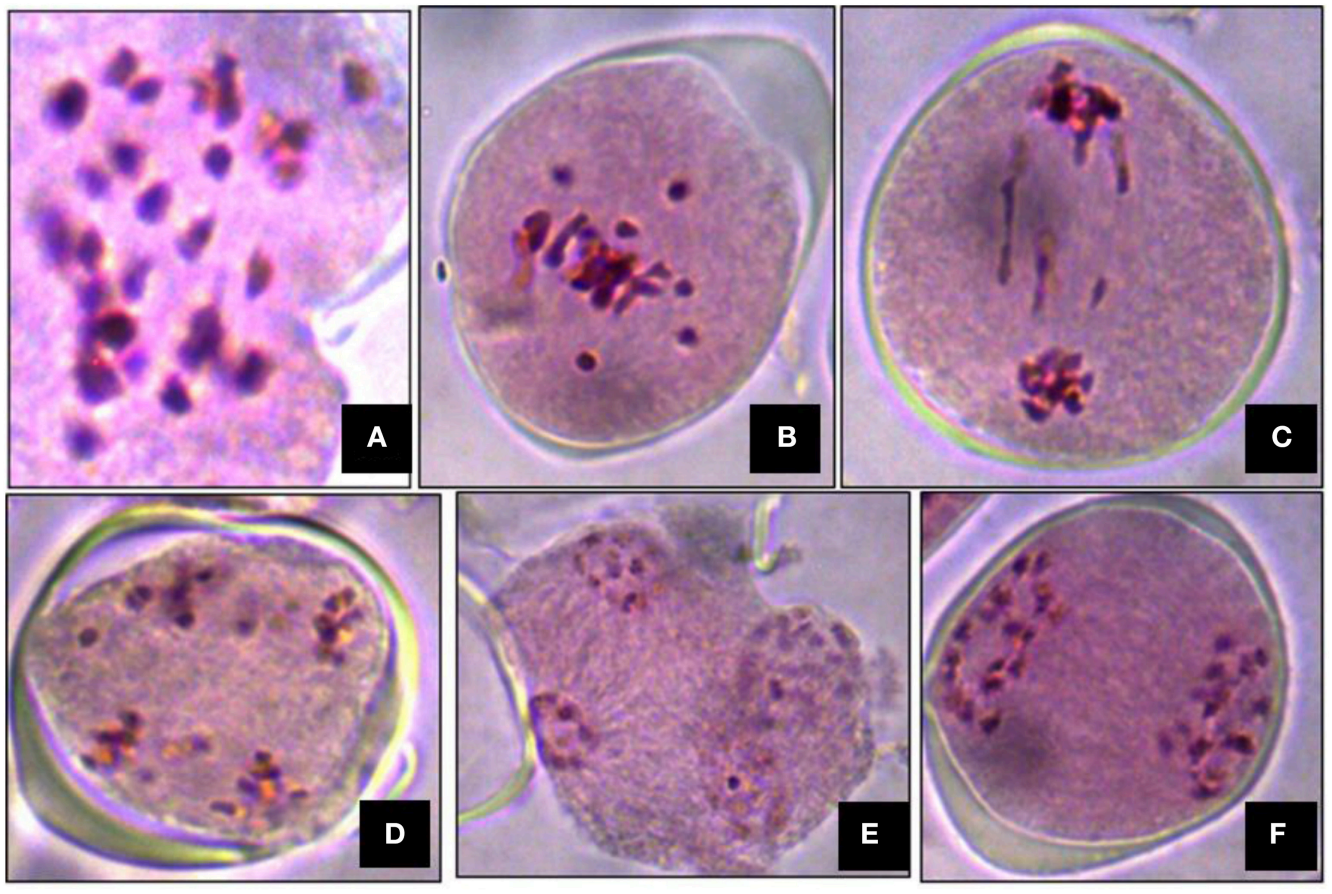
Cytological studies of inter-specific hybrids. Meiosis in the inter specific hybrid of *B. oleracea botrytis* group (2*n* = 18, CC) × *B. carinata* (2*n* = 4x = 34, BBCC). **(A)** The PMC at metaphase I showing 26 chromosomes. **(B)** Occurrence of univalents. **(C–E)** Irregular stages of anaphase I (presence of laggards in central part), early and late telophase II. **(F)** Regular meiosis in PMC.

**Figure 5:**
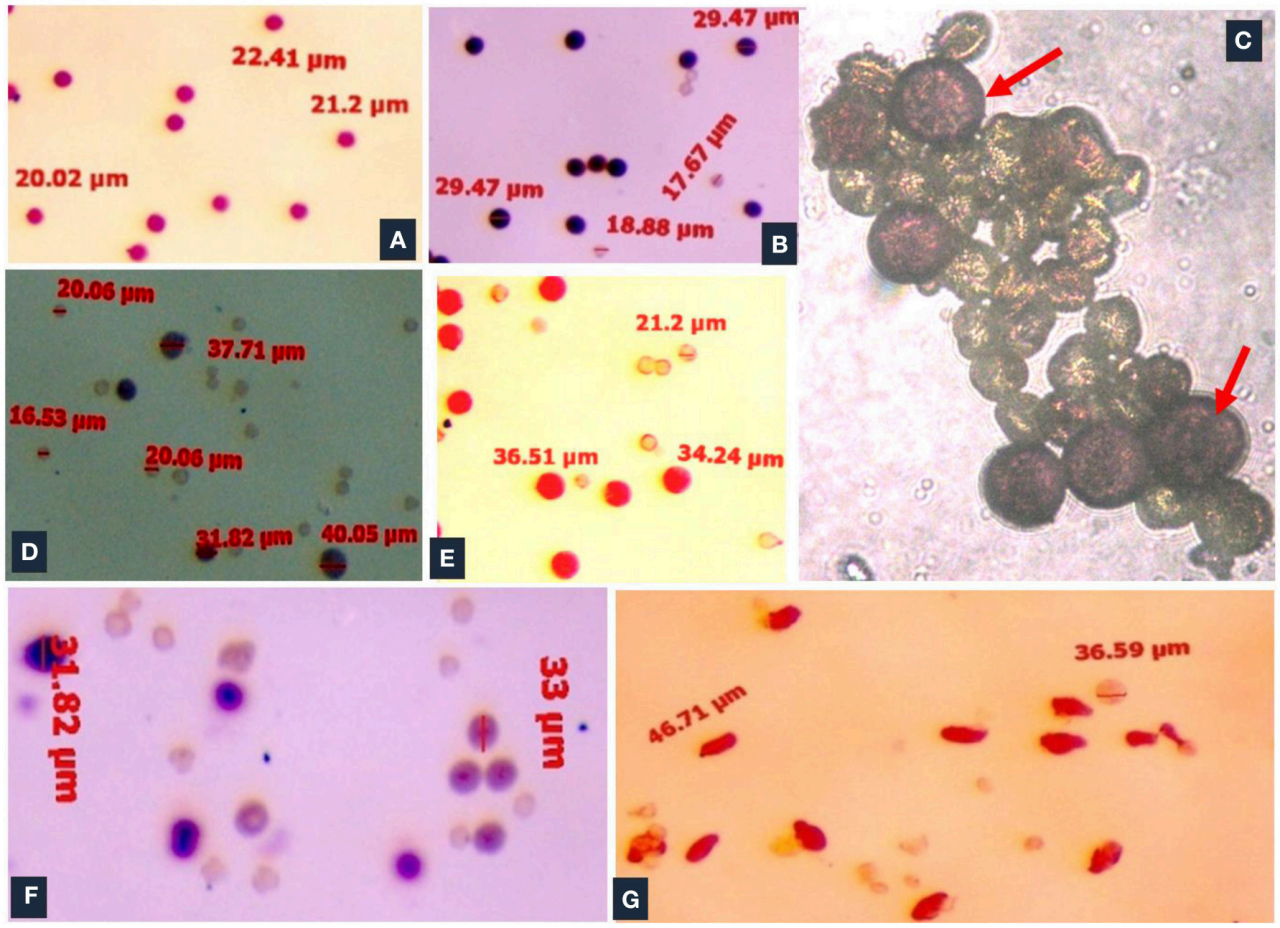
Pollen viability studies of inter-specific hybrids and BC_1_ population. **(A)** Cauliflower “Pusa Sharad.” **(B)** *B. carinata* “NPC-9.” **(C)** Frequent occurrence of pollen giant (red arrow) and others as normal pollen in F_1_ hybrids. **(D)** Poor pollen viability in inter-specific F_1_ hybrids. **(E–G)** BC_1_ individuals showed low to high pollen viabilty.

Meiosis in the di-genomic (BCC) interspecific hybrid of *B. oleracea botrytis* group (2*n* = 18, CC) × *B. carinata* (2*n* = 4x = 34, BBCC) was higly disorganized. The majority of PMCs of the interspecific hybrids showed 26 chromosomes at metaphase I (Figure 4A). There was preponderance of univalent in PMCs (Figure 4B). Anaphase I was highly irregular and there was presence of laggards in central part of PMCs (Figure 4C). Advance stages of meiosis like early and late telophase II (Figures 4D,E) were found showing irregular leads to production of unreduced gametes. A few number of PMCs were observed with regular anaphase I (Figure 4F). The mean pollen viability of interspecific hybrid was very low (2.77%) when compared with Pusa Sharad (98.00%) and “NPC-9” (93.33%) (Figures 5A,B,D). Average pollen viability (16.30%) of BC_1_ plants has been increased and observed in a range of 9.00 to 28.00% (Figures 5E–G). The average pollen size of Pusa Sharad (19.39 μm) and NPC-9 (27.34 μm) was observed and classified as normal pollen. However, pollen size of F_1_ interspecific hybrids was observed with variation in size from 10 to 19 μm to giant pollen (35–60 μm). Giant pollen grains, most likely formed from unreduced gametes, were more viable than normal pollen in F_1_ hybrid of Cauliflower × Ethiopian mustard (Figure 5C). Occurrence of giant pollen was also observed in BC_1_ showing variation in pollen size 35.0–70.67 μm.

### Phenotyping for black rot disease

**Figure 6:**
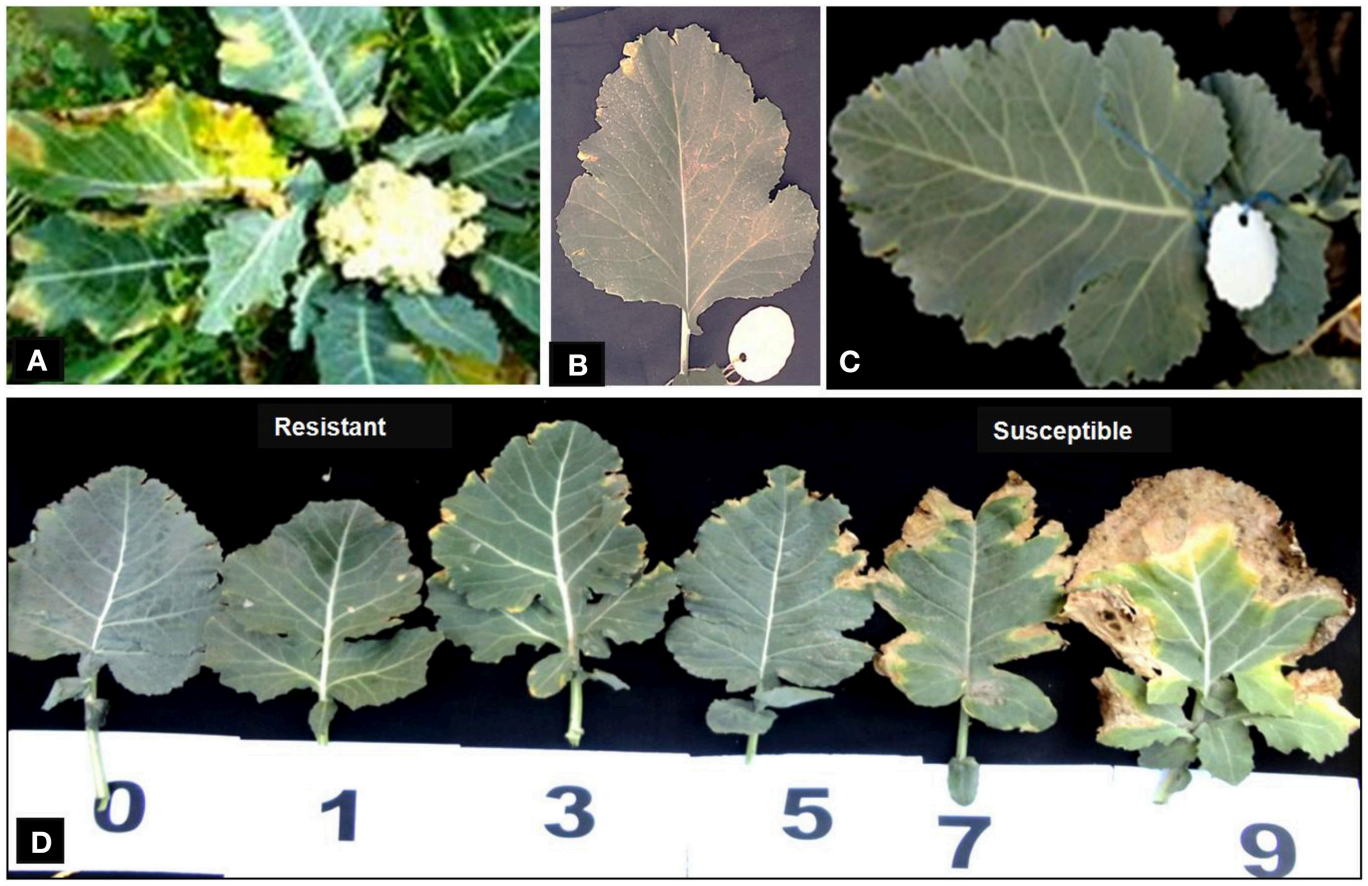
Phenotyping of introgressed BC_1_ for black rot disease. Disease reaction with (*Xcc* race 1) in parental genotypes. **(A)** Susceptible “Pusa Sharad.” **(B)** Resistant “NPC-9.” **(C)** All interspecific hybrid (resistant). **(D)** Variations for disease in BC_1_ plants.

Disease reaction including disease severity (0–9) and incidence (%) of parental genotypes, F_1_s and BC_1_ plants are given in Table 3. Cauliflower variety “Pusa Sharad” and *B. carinata* line “NPC-9” were found highly susceptible and resistant to *Xcc* race 1 (Figures 6A,B), respectively. All the six F_1_ hybrids were found to be highly resistant with average disease severity of 0.07 and 5.0 % incidence (Figure 6C). Thus, the resistance appears to be governed by single dominant genes in the *B. carinata* line, NPC-9 (Sharma et al., 2016). Variation for disease severity and incidence was observed in BC_1_ (Figure 6D) as among the 24 BC_1_ plants, 18 had strong resistance with low disease severity (0.03–0.88) and incidence (3.33–20.00%). Six BC_1_ plants were observed with high disease severity (3.84–8.77) and incidence (53.33–100%). All the susceptible plants started showing disease symptoms at 15 DAI and continuous growth of pathogen was observed.

**Table 3:** Phenotyping for black rot disease *Xcc* race 1 in parental, F_1_ and back crossed individuals derived from *in vitro* embryo rescue.

**Genotype**	**Disease incidence (%)**			**Disease severity (0–9)**			Disease reaction^*^
	15 DAI^*^	**21 DAI**	**30 DAI**	**15 DAI**	**21 DAI**	**30 DAI**	
Pusa sharad	43.33	100	100	3.00	4.60	8.77	Susceptible
NPC-9	0	0	3.33	0	0	0.03	Resistant
F_1_	0	5.00	5.00	0	0.07	0.07	Resistant
BC_1–1_	0	0	3.33	0	0	0.03	Resistant
BC_1–2_	0	3.33	3.33	0	0.03	0.03	Resistant
BC_1–3_	46.67	53.34	73.33	1.84	3.42	5.25	Susceptible
BC_1–4_	46.67	56.67	56.66	1.25	3.62	7.11	Susceptible
BC_1–5_	36.67	43.33	53.33	1.74	2.88	3.84	Susceptible
BC_1–6_	3.33	6.67	20.00	0.03	0.22	0.26	Resistant
BC_1–7_	0	0	10.00	0	0	0.11	Resistant
BC_1–8_	0	3.33	3.33	0	0.03	0.03	Resistant
BC_1–9_	0	6.67	10.00	0	0.07	0.11	Resistant
BC_1–10_	0	3.33	6.67	0	0.11	0.15	Resistant
BC_1–11_	60.00	73.33	100	2.44	6.44	8.77	Susceptible
BC_1–12_	43.33	60.00	70.00	2.40	4.51	6.40	Susceptible
BC_1–13_	66.67	83.33	100.00	4.07	7.10	7.58	Susceptible
BC_1–14_	0	0	10.00	0	0	0.18	Resistant
BC_1–15_	0	6.67	10.00	0	0.15	0.33	Resistant
BC_1–16_	40.00	56.67	63.33	1.70	2.14	5.32	Susceptible
BC_1–17_	3.33	6.67	6.67	0.03	0.07	0.07	Resistant
BC_1–18_	0	3.33	3.33	0	0.03	0.03	Resistant
BC_1–19_	10.00	13.33	16.67	0.11	0.15	0.26	Resistant
BC_1–20_	0	6.67	6.67	0	0.07	0.07	Resistant
BC_1–21_	3.33	10.00	13.33	0.03	0.11	0.22	Resistant
BC_1–22_	66.67	73.33	83.33	3.33	5.18	6.84	Susceptible
BC_1–23_	10.00	13.33	20.00	0.18	0.29	0.56	Resistant
BC_1–24_	13.33	20.00	20.00	0.18	0.51	0.88	Resistant

* *Disease reaction on the basis of disease severity (0–9) and disease incidence (%)*.

DAI^*^, Days after inoculation.

### Genotyping of BC_1_ population

**Figure 7:**
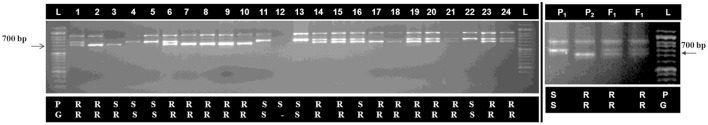
Genotyping of inter-specific BC_1_ population for black rot resistance using linked marker (ILPAt1g70610). (Resistance specific band ~700 bp), (1–24, BC_1_ plant number), P_1_, Pusa Sharad and P_2_, NPC-9, F_1_; Interspecific hybrid of “Pusa Sharad× NPC-9” P = phenotype for black rot disease, G = genotype for linked marker, R = resistant, S = susceptible and L; 1Kb DNA ladder. (Two gel pictures were grouped together and separated using black vertical line).

An ILPAt1g70610 marker linked with black rot resistance against *Xcc* race 1 in “NPC-9” of *Brassica carinata* (Sharma et al., 2016) was used for genotyping of BC_1_ population (Figure 7). This linked ILP marker (At1g70610) identified 75% individuals as resistant generating approximately 700 bp sized fragment and 20.83% individuals as susceptible in BC_1_ population. Out of these, only 4.17% could not amplify any band. However, 66.66 and 33.34% individuals in BC_1_ population were found resistant and susceptible upon phenotyping for black rot disease. Segregation distortion was observed for black rot resistance in inter-specific BC_1_generation. There were only 12.5% recombinants which were found to be genotypically resistant, however they were scored phenotypically susceptible. Successful introgression of black rot resistance locus from B-7 chromosome of *B. carinata* to *B. oleracea botrytis* group (Pusa Sharad) was confirmed by linked ILPAt1g70610 marker.

### Morphological characterization

**Figure 8:**
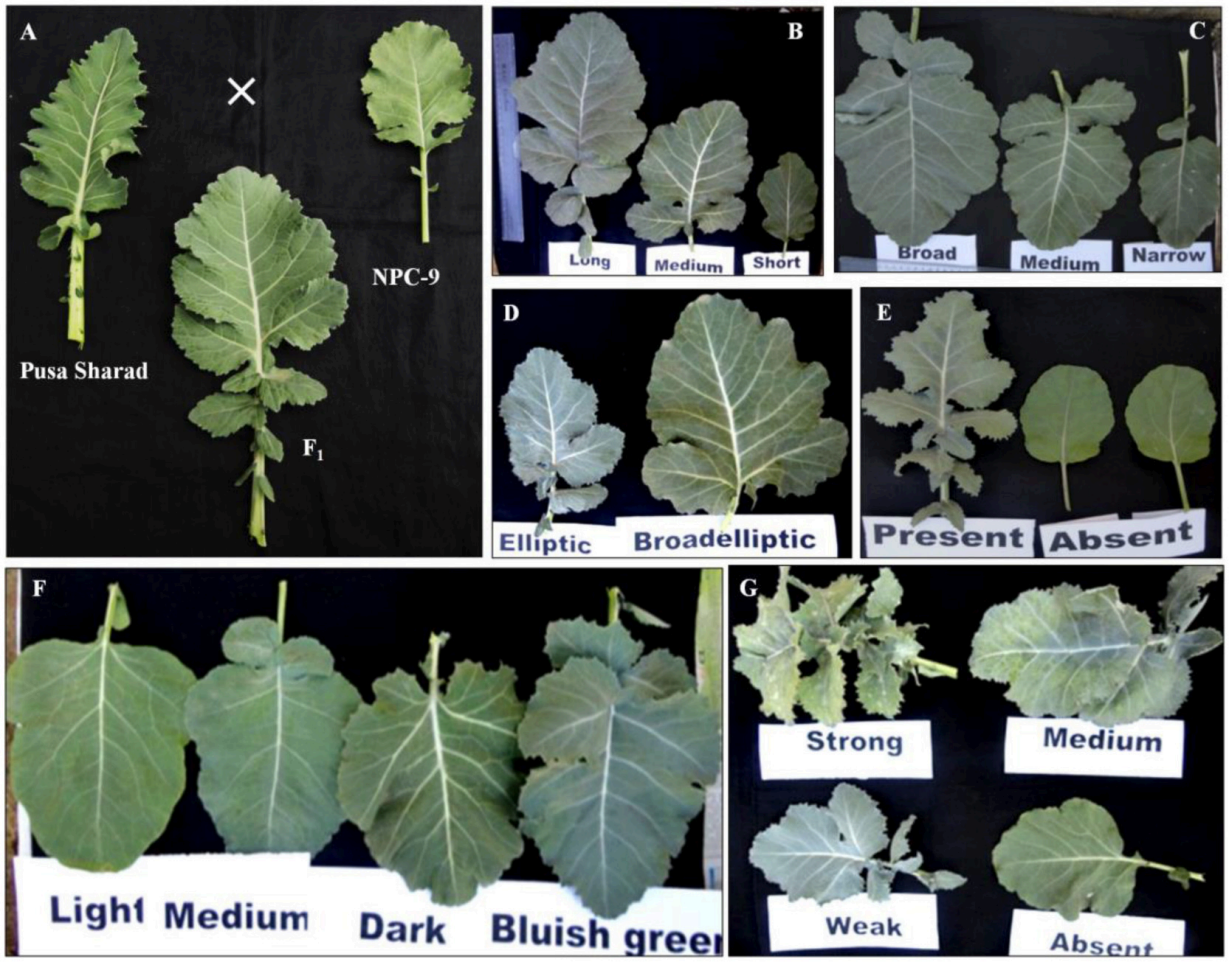
Leaf characteristics and morphology of parental genotypes with BC_1_ plants. **(A)** Cauliflower (Pusa Sharad), *B. carinata* (NPC-9), Inter-specific (F_1_) hybrid. **(B)** Leaf length. **(C)** Leaf width. **(D)** Leaf shape. **(E)** Leaf lobes. **(F)** Leaf color. **(G)** Leaf puckering variations in BC_1_.

**Figure 9:**
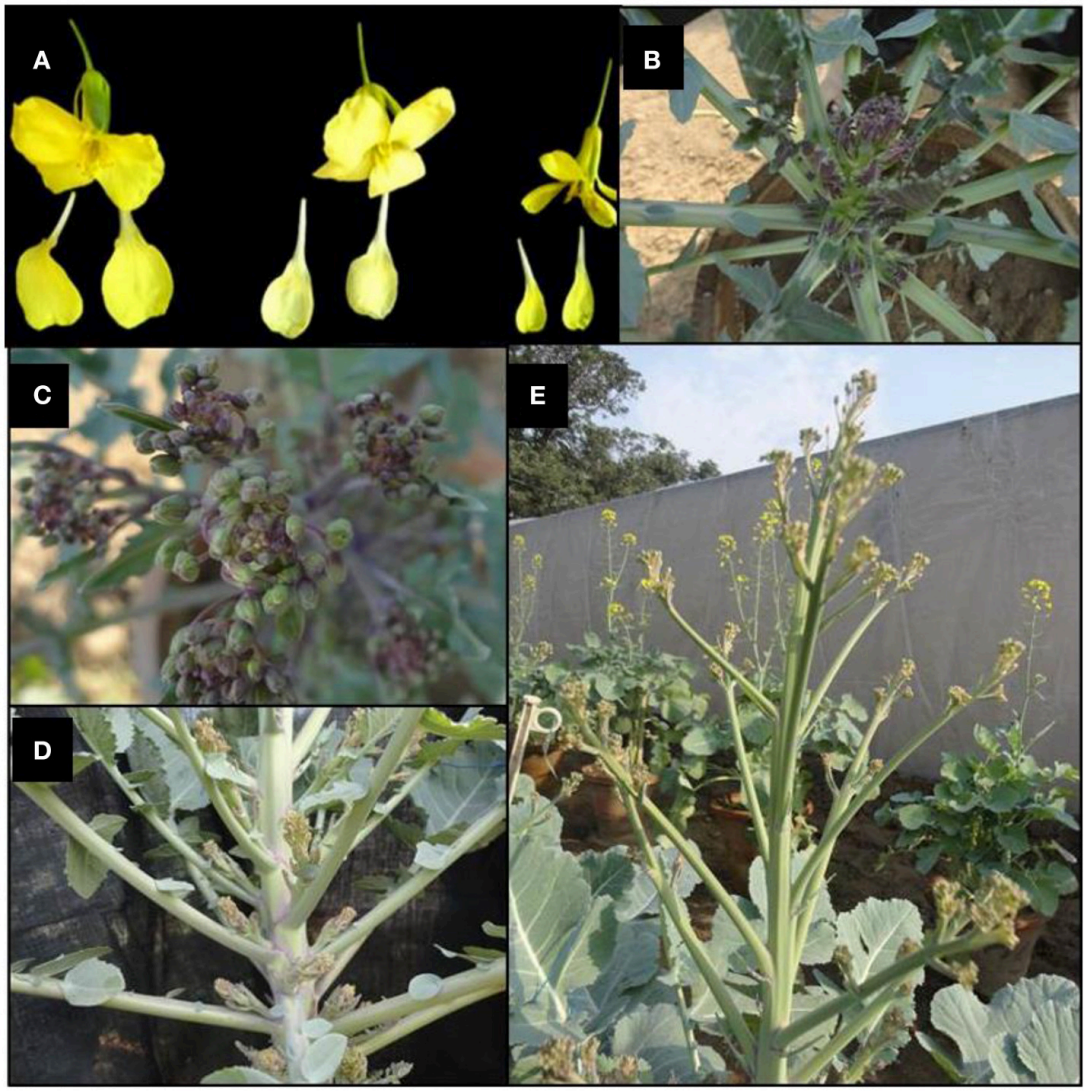
Morphological events of interspecific hybrid and BC_1_ progeny. **(A)** Different size (broad, medium, and narrow) and colors (yellow and cream light yellow) of petals (BC_1_). **(B)** Initiation of flower stalk and closer view **(C)** of inflorescence with purple variegation in interspecific hybrid. **(D)** Development of many swollen axillary buds prior to elongation of the flowering axis in interspecific hybrid. **(E)** Branched flowering stalk of an individual of BC_1_ population.

The F_1_ hybrids were grown to flowering. They were healthy and intermediate in most of their quantitative characters with respect to their parents. The parental genotypes, F_1_ and BC_1_ generations were characterized for 20 different morphological traits (Supplementary Table 2). Semi-erect leaf attitude was observed in 62.50% BC_1_ individuals, F_1_'s and Pusa Sharad. BC_1_ individuals exhibited variation in leaf length and width ranging from 8.1 to 31.2 cm and 5.2 to 20.1 cm, respectively. The F_1_ plants, however, had intermediate leaf length and width between 25.0–30.0 and 10–13 cm, respectively. All the F_1_ plants and majority of BC_1_ plants (62.50%) were observed with broad elliptic leaf shape unlike Pusa Sharad which had elliptic leaf shape. A few segregants (4.17%) in BC_1_ were observed which did not have leaf lobes, whereas all the F_1_ plants and parental genotypes had presence of leaf lobes. Leaf color was found bluish green in F_1_ plants which segregated as majority of backcross progeny bluish green (45.83%) leaf color like cauliflower resembling followed by light green (41.67%) and dark green (12.50%). Most of the individuals of BC_1_ (75.0%) and all inter-specific F_1_ plants showed medium waxiness pattern on leaves like that of cauliflower. Medium leaf puckering was visualized in 50% BC_1_ and all F_1_ plants like that of cauliflower, whereas leaf waxiness and puckering was completely absent in *B. carinata* “NPC-9.” Strong puckering (8.33%) and waxiness (4.17%), leathery appearance and strong mid vein thickness (4.17%) were also observed in BC_1_. Leaf apex was round (54.17%) like *B. carinata*, intermediate (4.17%) like F_1_'s and pointed (37.50 %) as in case of recurrent parent cauliflower. Mid vein thickness of leaf showed variation categorized as strong (4.17%), thick (16.66%), medium thick (75.0%) and very low (4.16%) in BC_1_, while all F_1_'s and Pusa Sharad had thick mid vein unlike *B. carinata* “NPC-9.” Serrate type leaf dentation was reported in majority of BC_1_ individuals (75.0%) resembling cauliflower and all F_1_'s unlike *B. carinata* “NPC-9” exhibited entire type. The leaf morphology was found to have indication toward cauliflower type in all the F_1_'s and majority of BC_1_ individuals. The plant height among F_1_ plants was found to be intermediate (100–110 cm) with thick stem, multiple lateral buds and short inter-nodal length unlike “NPC-9,” whereas the plant height of Pusa Sharad and “NPC-9” ranged between 80.0–85.0 and 168.0–178.0 cm, respectively. In BC_1_ individuals, the plant height varied significantly between 19.5 and 156.8 cm comprising dwarf, medium and tall plants. Flower petal color varied from creamy white (4.16%), light yellow (20.84%) to yellow (75.0%) in BC_1_ population, whereas F_1_'s exhibited yellow color like that of NPC-9 and Pusa Sharad had light yellow color petals. In BC_1_ population, flower stalk length showed wide variation ranging from 10.70 to 85.60 cm. The dwarf stunted plants, however, produced very dwarf flowering stalk. Interspecific F_1_'s gave intermediate length of flowering stalk (65–75 cm). Purple variegated flowering stalk color like Pusa Sharad was prominent (54.67%) followed by green color (45.83%) in BC_1_ plants, whereas all the F_1_'s had purple variegated stalk unlike “NPC-9.” The BC_1_ Plants exhibited variation in petal length and width ranging from 1.1–1.9 to 0.4–1.2 cm, respectively, whereas F_1_'s have medium length (1.5 cm) and broad (0.9 cm) width of petal. All the F_1_'s and BC_1_ plants produced short siliqua ranging from 3.0–3.2 to 1.2–3.8 cm, respectively unlike Pusa Sharad and NPC-9. Majority of BC_1_ plants (83.33%) produced open type siliqua angle with main shoot like that of cauliflower ‘Pusa Sharad’ followed by semi-appressed (12.5%) and appressed (4.16%), whereas all interspecific hybrids and NPC-9 had semi-appressed type siliqua angle. The entire F_1_ hybrid plants and most of the BC_1_ plants (83.33 %) bore smooth texture siliqua like that of cauliflower variety Pusa Sharad, while NPC-9 produced undulated texture siliqua. The morphological and floral traits events of interspecific hybrid and BC_1_ progeny are illustrated (Figures 8, 9).

## Discussion

Hybridization and backcrossing are normally employed for the introgression of desirable characters, especially biotic and abiotic stresses and quality traits. However, monitoring the outcomes of inter-varietal hybridization and related wild species, homeologous chromosome pairing and consequent gene transfer are pertinent issues. Hybridization between tetraploid and diploid species is cumbersome and leads to failures at many stages starting from pollination incompatibility due to pre/post-fertilization barriers. In the present study, there was no natural seed setting in F_1_ hybrid of “Pusa Sharad (*B. oleracea botrytis* group) × NPC-9” (*B. carinata*) and their BC_1_. However, some siliqua got enlarged as fully grown, but these contained shriveled, brown ovules only. Most of the interspecific pollinations did not produce any mature seeds due to failure of endosperm development. Similar findings have also been reported by earlier researchers (Diederichsen and Sacristan, 1994). Fertilization may take place, but abortion of embryos during early development stage (Weerakoon, 2011) may prevent natural seed setting. There have been several investigations which focused on improving the interspecific hybridization efficiency in *Brassica* (Rahman, 2004; Zhang et al., 2004), but the success rate had been reported to be very low particularly in crosses, where *B. oleracea* was used female parent (Stewart, 2002; Dey et al., 2015). In our study, the efficacy of two *in vitro* embryo rescue methods (siliqua and ovule culture) under two different temperature regimes was statistically at par. However, on the basis of average success rate (%), direct ovule culture was better and it was found significantly efficient (*p* = 0.02 < *p* = 0.05) in embryo rescue to produce BC_1_ compared to that of F_1._ Besides these methods of embryo rescue and growing temperature, the medium composition, growth hormone, number of cultured ovules/siliqua, ploidy of parental genotypes, accurate timing of embryo rescue and progeny of inter-specific cross (F_1_/BC_1_/BC_2_) might play a key role in the efficiency of embryo rescue of a wide cross. Optimum stages for siliqua culture were found to be 5–7 DAP and 9–11 DAP under low and high temperature regimes, respectively upon culturing for a week approximately. Whereas, Hansen and Earle (1995) could obtain best results when they harvested ovaries 3 days after pollination and cultured for 2 weeks. The success rate of direct ovule culture method was higher (4.41%) under high temperature regime (26.4°C/10.4°C) than low temperature regime period (19.4°C/4.3°C) (1.75%). Maximum efficiency of direct ovule culture at 16 DAP under temperature regime of 20°C/15°C in interspecific cross *B. oleracea* × *B. napus* was also reported by Bennett et al. (2008).

Over all from this study, direct ovule culture has been found to be best method of embryo rescue which exhibited higher average success rate (3.20%) than siliqua-ovule culture (1.98%). This encouraged us to follow direct ovule culture to produce BC_1_ plants with average success rate 9.60%. Several investigators compared the efficiency of these techniques for the production of various *Brassica* interspecific hybrids and reported that ovule culture was superior to other techniques, especially when *B. oleracea* was used as female (Takeshita et al., 1980). A significantly less number of embryos could be rescued at 21 DAP which suggested that degeneration of the hybrid embryos starts after 21 DAP. Increasing temperature has been found to be the cause of enhancement of embryo maturity. The optimum stage of the siliqua between 15 and 19 DAP was found to be most suitable for rescue of hybrid embryos via direct culture during low temperature regime. These findings were, however, contradictory to Rahman (2004) who reported that highest numbers of embryos were rescued between 20 and 28 DAP depending upon the specific cross undertaken. This difference may be due to various growing conditions and genotype. Based on the previous studies, stage of embryo rescue and success of inter-specific hybridization has been found associated with parental genotypes (Meng and Lu, 1993) and growing conditions of plant materials. During low temperature regime, early stage (11–13 DAP) ovules have been found to have more chances of getting damaged during embryo rescue and sensitivity to osmotic shock under *in vitro* culture. These findings are in line with those of Raghavan and Srivastava (1982). Culturing turgid ovules in dark decreases osmotic shock and advancement of embryo maturity.

During high temperature regime, 15 DAP was observed critical stage for direct ovule culture. Age of the siliqua between11 and 13 DAP was found to be most suitable for rescue of hybrid embryos *via* direct culture under high temperature regime. We faced problems in getting turgid ovules from later stage siliquae. It is assumed that high temperature coupled with prolonged duration may cause embryo degeneration. The successful application of this technique depends on the stage of the embryo being rescued and cultured *in vitro*. Introgression from alien *Brassica* species to *B. oleracea* has been accomplished for various other traits, such as powdery mildew (Tonguc and Griffiths, 2004a), downy mildew (Chiang et al., 1977), male sterility (Chiang and Crete, 1987) and atrazine resistance (Jourdan et al., 1989). The presence of anthocyanin pigmentation on anther tip of all interspecific F_1_ plants confirmed the hybridity and unveiled dominant nature of anthocyanin pigmentation in the present investigation. Similarly, Guo et al. (2012) also reported dominant nature of anthocyanin pigmentation on anther tip in *B. carinata*. A co-dominant SSR NI2-C01 molecular marker confirmed true hybridity of all inter-specific F_1_ plants.

The B genome specific primer (DB) amplified 350 bp size band in “NPC-9” (BBCC) and in all di-genomic hybrids (BCC) but not in C genome of cauliflower. The C genome specific Primer (DC), however, amplified 625 bp size in both the parents as well as all digenomic hybrids (BCC) revealing true interspecific hybridity. Similar amplification size of PCR product specific to both B and C genomes were in accordance with Yan et al. (2014). Di-genomic F_1_ hybrid (BCC) showed the expected number of chromosomes (2*n* = 26) at metaphase I. Due to frequent occurrence of univalents in PMCs and irregular anaphase I, a typical characteristic of interspecific cross is the formation of unreduced gametes. In our study of direct crossing of *B. oleracea* × *B. carinata* using embryo rescue, though, the pollen fertility was low (2.77%) in interspecific hybrids, which was improved substantially(9–28%) in BC_1_. On the contrary, Dey et al. (2015) reported F_1_ sterility which later showed slight fertility in BC_1_ progenies (0–4%) in the entire cross combinations of *B. oleracea botrytis* group × *B. carinata* as well as its reciprocal crosses. Sheng et al. (2012) reported semi-fertile interspecific hybrids of *B. rapa* × *B. nigra* with increased mean pollen fertility(28%) of BC_1_ along with high level of resistance against black rot disease.Whereas, Hansen and Earle (1995) reported fertile resistant hybrids developed by protoplast fusion between rapid-cycling *B. oleracea* and a highly resistant *B. napus* line to *X. campestris* pv. *campestris* in order to transfer resistance to *Xcc* identified in *B. carinata* (PI 199947: previously identified as *B. napus*) to *B. oleracea*.

Although, interspecific hybrid plants were male sterile from of *B. oleracea* × *B. juncea* accession number “A1982” (Tonguc and Griffiths, 2004b), but giant pollen (= 35 μm) in a cross combination of Cauliflower × Ethiopian mustard revealing more viability than normal size (18–20 μm) pollen grains in present study. Mason et al. (2011) also reported giant pollen in *B. napus* × *B. carinata, B. juncea* × *B. carinata* and *B. juncea* × *B. napus* cross combinations and suggested that unreduced gametes may be more viable. It is assumed that these giant pollens were most likely formed from unreduced gametes during irregular meiosis. Interspecific hybrids tend to produce greater frequencies of unreduced gametes than their parents (Ramsey and Schemske, 1998). These viable unreduced gametes would be readily available for evolution of polyploid species *via Brassica* interspecific hybrids, which may be explored to unveil the triploid bridge hypothesis of allopolyploid evolution.

High level of resistance to black rot was observed in all the F_1_ hybrid plants and some of the resistant plants did not exhibit any disease symptoms indicating that resistance source NPC-9 was homozygous and stable in the F_1_ progeny. Tonguc et al. (2003), however, observed some susceptible plants in F_1_ derived from resistant 11B-1-12 parent plants, suggesting that the resistance was heterozygous and/or not stable in the F_1_ progeny. In phenotyping (66.66%) and genotyping (75%) of BC_1_ individuals, strong resistance was observed against *Xcc* race 1 and the proportion of resistant plants was significantly higher as against normally expected (1R:1S).These findings are in line with those of Tonguc et al. (2003) and Wang et al. (2011). The probable causes of segregation distortion observed could be due to low success rate of embryo rescue, low BC_1_ population size (only 24), gametic abortion, unreduced gametes, and any chromosomal instability such as aneuploidy or unstable translocations. However, Tonguc and Griffiths (2004b) reported that all the interspecific hybrids between *Brassica oleracea* L. cultivars and *Brassica juncea* A 19182, and BC_1_ plants were resistant to black rot races 1 and 4.

The linked ILP marker identified 18 BC_1_ individuals as resistant and 5 as susceptible. One individual, however, could not amplify any band. Successful introgression of black rot resistance locus from B-7 chromosome of NPC-9 (*B. carinata*) to Pusa Sharad (*B. oleracea botrytis* group) was confirmed by linked ILPAt1g70610 marker. In our study segregation distortion was observed for black rot resistance in inter-specific BC_1_ generation and revealed 12.50% recombinants in 24 BC_1_ individuals. Crossing over between linked marker and resistance locus or deletions of chromosomal segments may be the cause of the recombination. Tonguc et al. (2003) found eight amplified polymorphic RAPD markers associated with disease free plants in the breeding line “11B-1-12” obtained from protoplast fusion between susceptible *B. oleracea* and resistant *B. carinata* accession (PI 199947). Twenty eight putative introgression lines (ILs) were preselected according to a series of morphological (leaf shape and color, plant height and branching, curd features, and flower traits) and physiological (black rot/club root resistance) characters from a somatic hybridization of cauliflower and black mustard (Wang et al., 2016).

Our trait of interest *Xcc* resistance locus (*Xca1bc*) located on B7 chromosome of *B. carinata* has more chances of intact inheritance generation after generation. Homoeology between C genome of *B. oleracea* and B genome *B. carinata* is high as compared to that of other species *B. juncea* (AABB) and *B. nigra* (BB) of B genome. Panjabi et al. (2008) also demonstrated some degree of homoeology between the B and A/C-genomes. It is, therefore, evident that transfer of single dominant (R) gene is feasible as recombination might occur between homeologus chromosomes of B and C genome, respectively as prerequisite for introgression and further backcrossing are necessary to create genotypes with 18 chromosomes of C genome and additional resistance to *Xcc* (Marthe et al., 2010).

Recently, blackleg resistance have been introgressed from *B. carinata* to *B. napus* and reported that four chromosomes B1, B3, B7, and B8 were retained as intact or broken, while other four B genome chromosomes B2, B4, B5, and B6 were eliminated in BC_2_S_3_ interspecific progenies (Fredua et al., 2014). Navabi et al. (2011) also reported that most of the B genome chromosomes in BC_3_S_1_ plants tended to be inherited as intact linkage groups, but loss of terminal segments or translocations were also detected in several cases. It will be necessary to stabilize this black rot resistance gene in *B. oleracea* genotypes for utilization in commercial breeding. One approach could be to examine meiotic behavior of resistant plants and select plants with most regular meiosis to produce selfed/DH and backcross generations. Repeated backcrossing with cauliflower would eliminate extra chromosomal fragments and stabilize the transmission of resistance. Although BC_1_ exhibited variation for morphological traits, however we selected cauliflower leaf resembling plants with high black rot resistance for further backcrossing with cauliflower “Pusa Sharad” as recurrent parent to produce BC_2_ progeny and selfing produce BC_1_S_1._

Although a range of phenotypic variation was observed in BC_1_, leaf morphology of BC_1_ plants was either intermediate to both parents or similar in appearance to the cauliflower. These results are in tune with those of Bennett et al. (2008). Many dominant parental-specific phenotypes viz., variegated flowering stalk, glossy leaves, round leaf apex, obvious midrib, and waxiness were observed. While some traits like leaf size, leaf width, leaf apex, plant height, and flower stalk length of hybrids exhibited morphological features that are intermediate between those of cauliflower and Ethiopian mustard. However, the leaf color was close to the cauliflower in F_1_. These results are in tune with those of Wen et al. (2008). Development of swollen axillar buds prior to elongation of the flowering axis were frequently observed in interspecific hybrids. Consecutively, reduction in plant height and flowering stalk was observed in F_1_ and BC_1_ plants. Some of the plants were found to be tall like that of *B. carinata*, whereas some were dwarf with stunted growth. There was segregation distortion for most of the leaf and floral traits in BC_1_.

## Conclusion

For introgressing monogenic dominant black rot resistance identified in NPC-9 of *Brassica carinata* the interspecific hybrids between *B. oleracea botrytis* group (Pusa Sharad) and *B. carinata* (NPC-9) and their first backcross progeny conferring high resistance to *Xcc* race 1 were successfully developed, characterized and resistance confirmed using closely linked marker to gene of interest. Successive backcrossing with cauliflower will be required to recover the C genome specific desirable horticultural traits. Besides, observation would be recorded systematically to know meiotic behavior of resistant plants and allow selection of most regular meiosis which will enable production of selfed/DH and backcross generation plants for stable introgression.

## Author contributions

PK conceived the research. BS and PK designed research experiment. BS, PK, and DS conducted experiments. BS, PK, DS, and TS analyzed the data and prepared the manuscript. All the authors have read and approved the final manuscript.

### Conflict of interest statement

The authors declare that the research was conducted in the absence of any commercial or financial relationships that could be construed as a potential conflict of interest.

## Abbreviations

DAP: Days after pollination
DAI: Days after inoculation
BAP: 6- Benzyl amino purine
MS: Murashige and Skoog medium
IBA: Indole-3-butyric acid
ILP: Intron Length Polymorphic
SSR: Simple Sequence Repeats
TTG: Transparent testa glabra
PCR: Polymerase chain reaction
*Xcc*: *Xanthomonas campestris* pv. *campestris*.

## References

[B1] Ayotte R. Harney P. M. Souza M. V. (1987).The transfer of atriazine resistance from *Brassica napus* L. to *B. oleracea* L. Production of F_1_ hybrids through embryo rescues. Euphytica 36, 615–24. 10.1007/BF00041511

[B2] Bennett R. Thiagarajah M. R. King J. R. Rahman M. H. (2008). Interspecific cross of *Brassica oleracea* var. alboglabra and B. napus: effects of growth condition and silique age on the efficiency of hybrid production and inheritance of erucic acid in the self-pollinated back cross generation. Euphytica 164, 593–601. 10.1007/s10681-008-9788-0

[B3] Chiang M. S. Chiang B. Y. Grant W. F. (1977).Transfer of resistance to race 2 of *Plasmodiophora brassicae* from *Brassica napus* to cabbage (*B. oleracea* var. *capitata*). I. Interspecific hybridization between *B. napus* and *B. oleracea* var. *capitata* Euphytica 26, 319–326. 10.1007/BF00026993

[B4] Chiang M. S. Crete R. (1987). Cytoplasmic male sterility in *Brassica oleracea* induced by *B*. napus cytoplasm-female fertility and restoration of male fertility. Can. J. Plant Sci. 672, 891–897. 10.4141/cjps87-126

[B5] Dey S. S. Sharma K. Dey R. B. Kumar G. M. S. Singh D. Kumar R. (2015). Inter specific hybridization (*Brassica carinata* × *Brassica oleracea*) for introgression of black rot resistance genes into Indian cauliflower (*B. oleracea* var. botrytis L.). Euphytica 204, 149–162. 10.1007/s10681-015-1352-0

[B6] Diederichsen E. Sacristan M. D. (1994). The use of ovule culture in reciprocal hybridization between *B. campestris* L. and *B. oleracea* L. Plant Breed. 113, 79–82. 10.1111/j.1439-0523.1994.tb00706.x

[B7] Enjalbert J. N. Zheng S. Johnson J. J. Mullen J. L. Byrne P. F. McKay J. K. (2013). Brassicaceae germplasm diversity for agronomic and seed quality traits under drought stress. Ind. Crops Prod. 47, 176–185. 10.1016/j.indcrop.2013.02.037

[B8] Fredua A. R. Coriton O. Huteau V. Parkin I. A. Chevre A. M. Rahman H. (2014). Molecular cytogenetic identification of B genome chromosomes linked to blackleg disease resistance in Brassica napus × B. carinata interspecific hybrids. Theor. Appl. Genet. 127, 1305–1318. 10.1007/s00122-014-2298-7 24687759

[B9] Guo S. Zou J. Li R. Long Y. Chen S. Meng J. (2012). A genetic linkage map of *Brassica carinata* constructed with a doubled haploid population. Theor. Appl. Genet. 125, 1113–1124. 10.1007/s00122-012-1898-3 22669300

[B10] Gupta S. C. Kapoor V. K. (2002). Fundamentals of Mathematical Statistics, 10th Edn. New Delhi: Sultan Chand and Sons.

[B11] Hansen L. N. Earle E. D. (1995). Transfer of resistance to *Xanthomonas campestris* pv. campestris into *Brassica oleracea* L. by protoplast fusion. Theor. Appl. Genet. 91, 1293–1300. 10.1007/BF00220944 24170061

[B12] Hayward A. C. (1993). The hosts of *Xanthomonas*, in Xanthomonas, eds Swings J. G. Civerolo E. L. (London: Chapman & Hall), 1–119.

[B13] Inomata N. (1993). Crossability and cytology of hybrid progenies in the cross between *Brassica campestris* and three wild relatives of *B. oleracea, B. bourgeaui, B. cretica and B. montana*. Euphytica 69, 7–17. 10.1007/BF00021721

[B14] Inomata N. (2002). A cytogenetic study of the progenies of hybrids between *Brassica napus* and *B. oleracea, B. bourgeaui, B. cretica* or *B. montana*. Plant Breed. 121, 174–176. 10.1046/j.1439-0523.2002.00695.x

[B15] Jourdan P. S. Earle E. D. Mutscher M. A. (1989). Atrazine-resistant cauliflower obtained by somatic hybridization between *Brassica oleracea* and ATR- *B. napus*. Theor. Appl. Genet. 78, 271–279. 10.1007/BF00288810 24227155

[B16] Kapoor K. S. Gill H. S. Sharma S. R. (1985). A technique for artificial inoculation of cauliflower seedlings with *Sclerotinia sclerotiorum* (Lib) de Bary. Phytopathology 112, 191–192. 10.1111/j.1439-0434.1985.tb04827.x

[B17] Leflon M. Eber F. Letanneur J. C. Chelysheva L. Coriton O. Huteau V.. (2006). Pairing and recombination at meiosis of *Brassica rapa* (AA) × *Brassica napus* (AACC) hybrids. Theor. Appl. Genet. 113, 1467–1480. 10.1007/s00122-006-0393-0 16983552

[B18] Marthe F. Richter K. Schrader O. Ryschka U. (2010). *Cabbage (Brassica oleracea)* with new resistance to black rot (*Xanthomonas campestris* pv. *campestris*) from black mustard (*B. nigra*). Available online at: https://www.hortigate.de/Apps/WebObjects/Hortigate.woa/spider/meta?infometa=42020

[B19] Mason A. S. Huteau V. Eber F. Coriton O. Yan G. Nelson M. N.. (2010). Genome structure affects the rate of autosyndesis and allosyndesis in AABC, BBAC and CCAB *Brassica* interspecific hybrids. Chromosome Res. 18, 655–666. 10.1007/s10577-010-9140-0 20571876

[B20] Mason A. S. Nelson M. N. Yan G. Cowling W. A. (2011). Production of viable male unreduced gametes in Brassica interspecific hybrids is genotype specific and stimulated by cold temperatures. BMC Plant Biol. 11:103. 10.1186/1471-2229-11-103 21663695 PMC3141635

[B21] Meng J. Lu M. (1993). Genotype effects of *Brassica napus* on its reproductive behaviour after pollination with *B. juncea*. Theor. Appl. Genet. 87, 238–242. 10.1007/BF00223771 24190219

[B22] Momotaz A. Kato M. Kakihara F. (1998). Production of inter-generic hybrids between *Brassica* and *Sinapis* species by means of embryo rescue techniques. Euphytica 103, 123–130. 10.1023/A:1018331528368

[B23] Murashige T. Skoog F. (1962). A revised medium for rapid growth and bioassays with tobacco tissue cultures. Physiol. Plantarum 15, 473–497. 10.1111/j.1399-3054.1962.tb08052.x

[B24] Murray M. G. Thompson W. F. (1980). Rapid isolation of high molecular weight plant DNA. Nuc. Acids Res. 8, 4321–4326. 10.1093/nar/8.19.4321 7433111 PMC324241

[B25] Navabi Z. K. Huebert T. Sharpe A. G. Carmel M. Bancroft I. Isobel Parkin A. P. (2013). Conserved microstructure of the Brassica B Genome of *Brassica nigra* in relation to homologous regions of *Arabidopsis thaliana, B. rapa* and *B. oleracea*. BMC Genomics 14:250. 10.1186/1471-2164-14-250 23586706 PMC3765694

[B26] Navabi Z. K. Stead K. E. Pires J. C. Xiong Z. Sharpe A. G. Parkin I. A. (2011). Analysis of B-genome chromosome introgression in inter-specific hybrids of *Brassica napus* × *B. carinata*. Genetics 187, 659–673. 10.1534/genetics.110.124925 21196520 PMC3063663

[B27] Niemann J. Wojciechowski A. Jedryczka M. Kaczmarek J. (2013). Interspecific hybridization as a tool for broadening the variability of useful traits in rapeseed (*Brassica napus* L.), in Proceedings of the VIth IS on Brassicas and XVIIIth Crucifer Genetics Workshop, eds Branca F. Tribulato A. (Catania: Acta Hort), 1005.

[B28] Nishi S. Wata J. K. Toda M. (1959). On the breeding of interspecific hybrids between two genomes. ‘C’ and ‘B’ of *Brassica* through the application of embryo culture techniques. Japan. J. Breed. 8, 215–222. 10.1270/jsbbs1951.8.215

[B29] Pan X. Caldwell C. D. Falk K. C. Lada R. (2012). The effect of cultivar, seeding rate and applied nitrogen on *Brassica carinata* seed yield and quality in contrasting environments. Can. J. Plant Sci. 92, 961–971. 10.4141/cjps2011-169

[B30] Panjabi P. Jagannath A. Bisht N. C. Pdmaja K. L. Sharma S. Gupta V.. (2008). Comparative mapping of *Brassica juncea* and *Arabidopsis thaliana* using Intron Polymorphism (IP) markers: homoeologous relationships, diversification and evolution of the A, B and C Brassica genomes. BMC Genomics 9:113. 10.1186/1471-2164-9-113 18315867 PMC2277410

[B31] Raghavan V. Srivastava P. S. (1982). Embryo culture, in Experimental Embryology of Vascular Plants, ed Johri B. M. (Berlin: Springer-Verlag), 195–230. 10.1007/978-3-642-67798-4_9

[B32] Rahman M. H. (2004). Optimum age of siliques for rescue of hybrid embryos from crosses between *Brassica oleracea, B. rapa* and *B. carinata. Can*. J. Plant Sci. 84, 965–969. 10.4141/P04-003

[B33] Ramsey J. Schemske D. W. (1998). Pathways, mechanisms, and rates of polyploidy formation in flowering plants. Annu. Rev. Ecol. Evol. Syst. 29, 467–401. 10.1146/annurev.ecolsys.29.1.467

[B34] Sharma B. B. Kalia P. Yadava D. K. Singh D. Sharma T. R. (2016). Genetics and molecular mapping of black rot resistance locus *Xca1bc* on Chromosome B-7 in Ethiopian Mustard (*Brassica carinata* A. Braun). PLoS ONE 11:e0152290. 10.1371/jo. 27023128 PMC4811439

[B35] Sheng X. Guiju W. Guo Y. Yan H. Zhao H. Liu F. (2012). A semi-fertile interspecific hybrid of *Brassica rapa* and *B. nigra* and the cytogenetic analysis of its progeny. Genet. Res. Crop. Evol. 59, 73–81. 10.1007/s10722-011-9669-6

[B36] Singh D. Dhar S. Yadava D. K. (2011). Genetic and pathogenic variability of Indian strains of *Xanthomonas campestris* pv. campestris causing black rot disease in crucifers. Curr. Microbiol. 63, 551–560. 10.1007/s00284-011-0024-0 21956666

[B37] Singh D. Rathaur P. S. Vicente J. G. (2016). Characterization, genetic diversity and distribution of *Xanthomonas campestris* pv. *campestris* races causing black rot disease in cruciferous crops of India. Plant Pathol. 65, 1411–1418. 10.1111/ppa.12508

[B38] Stewart A. V. (2002). A review of Brassica species, cross-pollination and implications for pure seed production in New Zealand. Agron N. Z. 32, 63–82.

[B39] Takeshita M. Kato M. Tokumasu S. (1980). Application of ovule culture to the production of intergeneric or interspecific hybrids in *Brassica* and *Raphanus*. Jpn. J. Genet. 55, 373–387. 10.1266/jjg.55.37312834064

[B40] Taylor I. D. Conway S. Roberts S. J. Astley D. Vicente I. G. (2002). Sources and origin of resistance to *Xanthomonas campestris* pv. *campestris* in *Brassica* genomes. Phytopathology 92, 105–111. 10.1094/PHYTO.2002.92.1.105 18944146

[B41] Tonguc M. Earle E. D. Griffiths P. D. (2003). Segregation distortion of *Brassica carinata* derived black rot resistance in *Brassica oleracea*. Euphytica 134, 269–276. 10.1023/B:EUPH.0000004947.37512.92

[B42] Tonguc M. Griffiths P. D. (2004b). Development of black rot resistant interspecific hybrids between *Brassica oleracea* L. cultivars and *Brassica* accession A 19182, using embryo rescue. Euphytica 136, 313–318. 10.1023/B:EUPH.0000032733.47031.5f

[B43] Tonguc M. Griffiths P. D. (2004a). Transfer of powdery mildew resistance from *Brassica carinata* to *Brassica oleracea* through embryo rescue. Plant Breed. 123, 587–589. 10.1111/j.1439-0523.2004.00987.x

[B44] Tonu N. N. Doullah M. A. Shimizu M. Karim M. M. Kawanabe T. Fujimoto R. (2013). Comparison of positions of QTLs conferring resistance to *Xanthomonas campestris* pv. *campestris* in *Brassica oleracea*. Amer. J. Plant Sci. 4, 11–20. 10.4236/ajps.2013.48A002

[B45] Vicente J. G. Holub E. B. (2013). *Xanthomonas campestris* pv. *campestris* (cause of black rot of crucifers) in the genomic era is still a worldwide threat to *Brassica* crops. Mol. Plant Pathol. 14, 2–18. 10.1111/j.1364-3703.2012.00833.x 23051837 PMC6638727

[B46] Vicente J. G. Taylor J. D. Sharpe A. G. Parkin I. A. Lydiate P. D. J. King G. J. (2002). Inheritance of race-specific resistance to *Xanthomonas campestris pv*. campestris in *Brassica* genomes. Phytopathology 92, 1134–1141. 10.1094/PHYTO.2002.92.10.1134 18944224

[B47] Wang G. L. V. J. Zhang J. Han S. Zong M. Guo N.. (2016). Genetic and epigenetic alterations of *Brassica nigra* introgression lines from somatic hybridization: a resource for cauliflower improvement. Front. Plant Sci. 7:1258. 10.3389/fpls.2016.01258 27625659 PMC5003894

[B48] Wang G. X. Tang Y. Yan H. Sheng X. G. Hao W. W. Zhang L.. (2011). Production and characterization of interspecific somatic hybrids between *Brassica oleracea* var. *botrytis* and *B. nigra* and their progenies for the selection of advanced pre-breeding materials. Plant Cell Rep. 30, 1811–1821. 10.1007/s00299-011-1088-9 21603996

[B49] Warwick S. I. (2011). Brassicaceae in agriculture, in Genetics and genomics of the Brassicaceae, eds Bancroft I. Schmidt R. (New York, NY: Springer), 33–67. 10.1007/978-1-4419-7118-0_2

[B50] Weerakoon S. R. (2011). Producing inter-specific hybrids between *Brassica juncea* (L.) Czern & Coss and *B. oleracea* (L.) to synthesize trigenomic (ABC) Brassica. J. Sci. Univ. Kelaniya 6, 13–34. 10.4038/josuk.v6i0.4218

[B51] Weerakoon S. R. Si P. Zili W. Meng J. Yan G. (2009). Production and confirmation of hybrids through interspecific crossing between tetraploid *B. juncea* and diploid *B. oleracea* towards a hexaploid Brassica population, in 16th Australian Research Assembly on Brassicas, (Ballarat, VIC).

[B52] Wen J. X. Li Z. Y. Fu T. D. Ma C. Z. Shen J. X. (2008). Improving ovary and embryo culture techniques for efficient resynthesis of *Brassica napus* from reciprocal crosses between yellow-seeded diploids *B. rapa* and *B. oleracea*. Euphytica 162, 81–89. 10.1007/s10681-007-9566-4

[B53] Westman A. L. Kresovich S. Dickson M. H. (1999). Regional variation in *Brassica nigra* and other weedy crucifers for disease reaction to *Alternaria brassicicola* and *Xanthomonas campestris* pv. campestris. Euphytica 106, 253–259. 10.1023/A:1003544025146

[B54] Yan M. Liu X. Chunyun G. Liu L. Xiang J. Ying L.. (2014). Cloning of *TTG1* gene and PCR identification of genomes A, B and C in *Brassica* species. Genetica 142, 169–176. 10.1007/s10709-014-9764-7. 24752509

[B55] Zhang G. Q. Tang G. X. Song W. J. Zhou W. J. (2004). Resynthesizing *Brassica napus* from interspecific hybridization between *Brassica rapa* and *B. oleracea* through ovary culture. Euphytica 140, 181–187. 10.1007/s10681-004-3034-1

[B56] Zhang G. Q. Zhou W. J. Guo H. H. Song W. J. Momoh E. J. J. (2003). Plant regeneration from the hybridization of *Brassica juncea* and *Brassica napus* through embryo culture. J. Agron. Crop Sci. 189, 1–4. 10.1046/j.1439-037X.2003.00059.x

[B57] Zhou Z. Weeden N. F. Dickson M. H. (1997). The expression of a resistant gene to black rot in progeny of the protoplast fusion broccoli (*B. oleracea*). Cruciferae Newsl. 19, 109–110.

