# Supplementary material for: Introgression of Black Rot Resistance from Brassica carinata to Cauliflower (Brassica oleracea botrytis Group) through Embryo Rescue
Source: Front Plant Sci. 2017 Jul 18;8:1255. doi: 10.3389/fpls.2017.01255 (PMC5513967; doi:10.3389/fpls.2017.01255)
Supplement: Supplementary file 1 [file Table1.DOCX]

Supplementary table 1. Sequences of DB, DC and SSR primes used for hybridity confirmation in this study

| Primer Id | Forward sequence (5´-3´) | Reverse Sequence (5´-3´) | PCR product  Size (bp) |
| --- | --- | --- | --- |
| DB | GACTCCTCCTCAACAACATCGAC | GAGCCTCAAGAGCGGCGTAT | 350 |
| DC | ACTCCGACTCCATGTCCCTCA | ACACTCCCCTGGTGCCTTTCA | 625 |
| SSR NI2-C01 | GAGTATGAGAGATGGGAATCCG | GACTGAGCAGCTTGGAGACC | 300, 225 |
