# Supplementary material for: Introgression of Black Rot Resistance from Brassica carinata to Cauliflower (Brassica oleracea botrytis Group) through Embryo Rescue
Source: Front Plant Sci. 2017 Jul 18;8:1255. doi: 10.3389/fpls.2017.01255 (PMC5513967; doi:10.3389/fpls.2017.01255)
Supplement: Supplementary file 2 [file Table2.DOC]

| Traits | Leaf: Attitude | Leaf: Length  (cm) | Leaf: Width  (cm) | Leaf: Shape | Leaf: Lobes | Leaf: Colour | Leaf: Waxiness | Leaf: Puckering | Leaf: Apex | Leaf: Mid-vein thickness | Leaf: Dentations of margin |
| --- | --- | --- | --- | --- | --- | --- | --- | --- | --- | --- | --- |
| Pusa Sharad | Semi erect | (35-50) | Medium  (19 ) | Elliptic | Present | Bluish green | Medium | Medium | Pointed | Prominent & thick | Serrate |
| NPC-9 | Horizontal | (25-35) | Broad (10) | Broad elliptic | Present | Dark green | Absent | Absent | Round | Less prominent & thin | Entire |
| F_1_ | Semi erect | (25-30) | Medium  (10-13) | Broad elliptic | Present | Bluish green | Medium | Medium | Semi round | Prominent & thick | Serrate |
| BC_1-1_ | Semi erect | 31.20 | 19.20 | Elliptic | Present | Bluish green | Medium | Strong | Pointed | Prominent & thick | Serrate |
| BC_1-2_ | Semi erect | 17.30 | 12.20 | Elliptic | Present | Bluish green | Medium | Medium | Semi round | Prominent & thick | Serrate |
| BC_1-3_ | Horizontal | 29.30 | 20.10 | Broad elliptic | Present | Dark green | Strong | Strong | Round | Prominent & Strong thick | Serrate |
| BC_1-4_ | Semi erect | 17.00 | 12.00 | Broad elliptic | Present | Light green | Light | Weak | Round | Prominent & thick | Dentate |
| BC_1-5_ | Horizontal | 18.30 | 13.00 | Broad elliptic | Present | Light green | Light | Weak | Round | Prominent & thick | Entire |
| BC_1-6_ | Horizontal | 29.10 | 18.30 | Broad elliptic | Present | Dark green | Light | Weak | Round | Prominent & thick | Dentate |
| BC_1-7_ | Semi erect | 26.20 | 15.40 | Broad elliptic | Present | Bluish green | Medium | Medium | Semi round | Prominent & thick | Serrate |
| BC_1-8_ | Horizontal | 21.80 | 11.70 | Broad elliptic | Present | Light green | Medium | Medium | Pointed | Prominent & thick | Dentate |
| BC_1-9_ | Semi erect | 27.00 | 18.00 | Broad elliptic | Present | Bluish green | Medium | Medium | Pointed | Prominent & thick | Serrate |
| BC_1-10_ | Horizontal | 22.13 | 11.10 | Elliptic | Present | Light green | Medium | Weak | Pointed | Prominent & thick | Serrate |
| BC_1-11_ | Semi erect | 30.10 | 19.30 | Broad elliptic | Present | Light green | Medium | Weak | Round | Prominent & thick | Dentate |
| BC_1-12_ | Semi erect | 23.15 | 16.30 | Broad elliptic | Present | Dark green | Light | Medium | Round | Prominent & thick | Dentate |
| BC_1-13_ | Semi erect | 8.10 | 5.00 | Irregular | Absent | Light green | Absent | Absent | Round | Less prominent & thin | Entire |
| BC_1-14_ | Horizontal | 25.10 | 12.30 | Broad elliptic | Present | Light green | Medium | Weak | Round | Prominent & thick | Serrate |
| BC_1-15_ | Semi erect | 18.20 | 10.70 | Elliptic | Present | Bluish green | Medium | Medium | Pointed | Prominent & thick | Serrate |
| BC_1-16_ | Horizontal | 27.40 | 18.30 | Broad elliptic | Present | Light green | Medium | Weak | Round | Prominent & thick | Serrate |
| BC_1-17_ | Semi erect | 25.00 | 13.10 | Broad elliptic | Present | Bluish green | Medium | Medium | Round | Prominent & thick | Serrate |
| BC_1-18_ | Semi erect | 16.90 | 11.70 | Elliptic | Present | Bluish green | Medium | Medium | Pointed | Prominent & thick | Serrate |
| BC_1-19_ | Horizontal | 19.70 | 10.20 | Broad elliptic | Present | Light green | Medium | Weak | Round | Prominent & thick | Serrate |
| BC_1-20_ | Semi erect | 19.20 | 12.30 | Broad elliptic | Present | Bluish green | Medium | Medium | Round | Prominent & thick | Serrate |
| BC_1-21_ | Semi erect | 29.40 | 17.00 | Elliptic | Present | Bluish green | Medium | Medium | Pointed | Prominent & thick | Serrate |
| BC_1-22_ | Horizontal | 21.00 | 11.00 | Broad elliptic | Present | Light green | Medium | Weak | Round | Prominent & thick | Serrate |
| BC_1-23_ | Semi erect | 16.90 | 10.00 | Elliptic | Present | Bluish green | Medium | Medium | Pointed | Prominent & thick | Serrate |
| BC_1-24_ | Semi erect | 25.17 | 17.10 | Elliptic | Present | Bluish green | Medium | Medium | Pointed | Prominent & thick | Serrate |

**Table 2: Morphological characterization of parental genotypes, interspecific F_1_ hybrid and backcross (BC_1_) plants developed through *In Vitro* embryo rescue**

Continue

| Traits | Flower: Colour of petals | Flower: Stalk length  (cm) | Flower: Stalk colour | Flower: Petal  Length (cm) | Flower: Petal width  (cm) | Siliqua: Length  (cm) | Siliqua: Angle with main shoot | Siliqua: Texture | Pollen  Viability (%) | Plant Height  (cm) |
| --- | --- | --- | --- | --- | --- | --- | --- | --- | --- | --- |
| Pusa Sharad | Light Yellow | Short (50) | Purple Variegated | Medium (1.4) | Medium(0.7) | Long (6.3) | Open | Smooth | 98.00 | Medium (82 ) |
| NPC-9 | Yellow | Long (90) | Green | Long (1.9) | Medium (0.6) | Medium  (5.1) | Semi Appressed | Undulated | 93.33 | 168 |
| F_1_ | Yellow | Medium (67) | Purple Variegated | Medium (1.5) | Broad (0.9) | Short (2.8) | Semi Appressed | Smooth | 2.77 | Medium (105) |
| BC_1-1_ | Yellow | 58.10 | Purple Variegated | 1.9 | 1.2 | 2.9 | Open | Undulated | 11.66 | 98.50 |
| BC_1-2_ | Yellow | 48.90 | Purple Variegated | 1.3 | 0.6 | 2.1 | Open | Smooth | 21.66 | 77.30 |
| BC_1-3_ | Yellow | 10.00 | Green | 1.0 | 0.3 | 1.0 | Appressed | Smooth | 15.66 | 19.00 |
| BC_1-4_ | Light Yellow | 41.30 | Green | 1.0 | 0.5 | 3.2 | Open | Undulated | 24.00 | 84.00 |
| BC_1-5_ | Light Yellow | 76.10 | Green | 1.9 | 0.9 | 3.6 | Open | Smooth | 12.66 | 130.00 |
| BC_1-6_ | Light Yellow | 65.30 | Green | 1.6 | 0.6 | 3.1 | Open | Smooth | 19.33 | 103.40 |
| BC_1-7_ | Yellow | 44.90 | Purple Variegated | 1.5 | 0.7 | 2.4 | Semi Appressed | Smooth | 18.33 | 90.30 |
| BC_1-8_ | Light Yellow | 37.40 | Green | 1.2 | 0.4 | 3.1 | Open | Smooth | 10.00 | 90.20 |
| BC_1-9_ | Yellow | 35.70 | Purple Variegated | 1.4 | 0.6 | 2.4 | Open | Smooth | 15.00 | 68.70 |
| BC_1-10_ | Yellow | 46.80 | Green | 1.8 | 1.2 | 2.5 | Open | Smooth | 23.66 | 81.70 |
| BC_1-11_ | Creamy White | 85.60 | Green | 1.8 | 0.9 | 3.8 | Open | Undulated | 28.00 | 156.60 |
| BC_1-12_ | Yellow | 41.30 | Green | 1.9 | 1.2 | 2.5 | Open | Smooth | 28.00 | 78.10 |
| BC_1-13_ | Yellow | 11.10 | Green | 1.0 | 0.2 | 1.0 | Open | Smooth | 14.33 | 21.10 |
| BC_1-14_ | Yellow | 76.70 | Purple Variegated | 1.9 | 1.3 | 3.7 | Open | Smooth | 14.33 | 115.70 |
| BC_1-15_ | Yellow | 44.80 | Purple Variegated | 1.7 | 0.8 | 2.9 | Open | Smooth | 16.33 | 78.20 |
| BC_1-16_ | Yellow | 50.00 | Green | 1.5 | 0.7 | 3.0 | Semi Appressed | Undulated | 27.33 | 85.70 |
| BC_1-17_ | Yellow | 64.70 | Purple Variegated | 1.5 | 0.6 | 3.8 | Open | Smooth | 12.33 | 114.20 |
| BC_1-18_ | Yellow | 49.70 | Purple Variegated | 1.4 | 0.6 | 3.1 | Open | Smooth | 11.33 | 80.70 |
| BC_1-19_ | Yellow | 57.30 | Purple Variegated | 1.3 | 0.5 | 3.0 | Open | Smooth | 14.67 | 91.80 |
| BC_1-20_ | Yellow | 62.50 | Purple Variegated | 1.4 | 0.7 | 3.1 | Open | Smooth | 13.00 | 95.70 |
| BC_1-21_ | Yellow | 78.50 | Purple Variegated | 1.8 | 0.9 | 3.7 | Open | Smooth | 9.33 | 110.73 |
| BC_1-22_ | Yellow | 47.00 | Green | 1.3 | 0.7 | 2.9 | Open | Smooth | 9.66 | 90.78 |
| BC_1-23_ | Yellow | 53.00 | Purple Variegated | 1.4 | 0.6 | 2.8 | Open | Smooth | 9.00 | 75.80 |
| BC_1-24_ | Light Yellow | 65.00 | Purple Variegated | 1.4 | 0.7 | 3.0 | Open | Smooth | 11.66 | 95.70 |
